# Evolution and origin of bread wheat

**DOI:** 10.1093/plcell/koac130

**Published:** 2022-05-04

**Authors:** Avraham A Levy, Moshe Feldman

**Affiliations:** Department of Plant and Environmental Sciences, Weizmann Institute of Science, Rehovot, 76100 Israel; Department of Plant and Environmental Sciences, Weizmann Institute of Science, Rehovot, 76100 Israel

## Abstract

Bread wheat (*Triticum aestivum,* genome BBAADD) is a young hexaploid species formed only 8,500–9,000 years ago through hybridization between a domesticated free-threshing tetraploid progenitor, genome BBAA, and *Aegilops tauschii*, the diploid donor of the D subgenome. Very soon after its formation, it spread globally from its cradle in the fertile crescent into new habitats and climates, to become a staple food of humanity. This extraordinary global expansion was probably enabled by allopolyploidy that accelerated genetic novelty through the acquisition of new traits, new intergenomic interactions, and buffering of mutations, and by the attractiveness of bread wheat’s large, tasty, and nutritious grain with high baking quality. New genome sequences suggest that the elusive donor of the B subgenome is a distinct (unknown or extinct) species rather than a mosaic genome. We discuss the origin of the diploid and tetraploid progenitors of bread wheat and the conflicting genetic and archaeological evidence on where it was formed and which species was its free-threshing tetraploid progenitor. Wheat experienced many environmental changes throughout its evolution, therefore, while it might adapt to current climatic changes, efforts are needed to better use and conserve the vast gene pool of wheat biodiversity on which our food security depends.

## Teaser

We describe the evolution of bread wheat in nature and under human selection with an emphasis on the donors of its subgenomes, evolution under polyploidy, and the “where when and how” of its domestication.

## Introduction

Bread wheat (*Triticum aestivum* L.), also known as common wheat, is an annual, predominantly autogamous species belonging to the Triticeae tribe of the grasses (Poaceae) family. It is an allohexaploid species, composed of 21 chromosome pairs organized in three subgenomes, A, B, and D, Genome BBAADD, 2*n* = 6*x* = 42 (Sears, 1952). Its nutritious grain contributes an important fraction of the calorific daily intake in many parts of the world, for example, 40%–50% in Egypt and Turkey and ∼20% in the UK (Shewry and Hey, 2015). It is also an important source of protein, dietary fibers, B vitamins, minerals, and other phytochemicals in the human diet. Starting with domestication in the fertile crescent, its cultivation spread worldwide in an unrivaled range of locations as far north as 67° N in Norway, Finland, and Russia, and south to 45° S in Argentina, being mostly grown in temperate regions and to a lesser extent also in sub-tropical and tropical habitats (Feldman, 2001). In 2020 and 2021, worldwide annual wheat production reached a record of ∼770 mt (FAO, 2021) with China and India as the top two producers. Once considered the food of western civilizations, wheat became one of the most important staple foods of humanity. Therefore, the question of wheat origin and evolution has fascinated humans since ancient times. Mac Key classified *T. aestivum* as having five subspecies, two hulled, ssp. *spelta,* ssp. *macha*, and three free threshing, ssp. ae*stivum*, ssp. *Compactum*, and ssp. *sphaerococcum* (Mac Key, 1954). All subspecies are domesticated, except an additional one, for one semi-wild form, ssp. *tibetanum*, presumably a feral wheat that escaped cultivation in Tibet (Shao et al., 1983). Here, we focus on *T. aestivum* ssp. *aestivum*, which is of global importance, the other subspecies being only locally cultivated, and we refer to it as bread wheat. In this review, we follow Mac Key’s classification, yet additional classifications were proposed as summarized by Sharma et al. (2021).

Despite the sequencing of its large approximately 16,000 Mbp genome (IWGS, 2018) and recent advances, major questions regarding bread wheat evolution remain: Which species are its progenitors? What was the impact of hybridization and allopolyploidization on the genome structure and stability? What were the main genetic changes during recent evolution under cultivation, domestication, and modern breeding? We present our current understanding of wheat evolution, starting from early discoveries to recent achievements and we discuss open questions.

## The diploid and tetraploid donors of wheat subgenomes and cytoplasm

**Figure 1:**
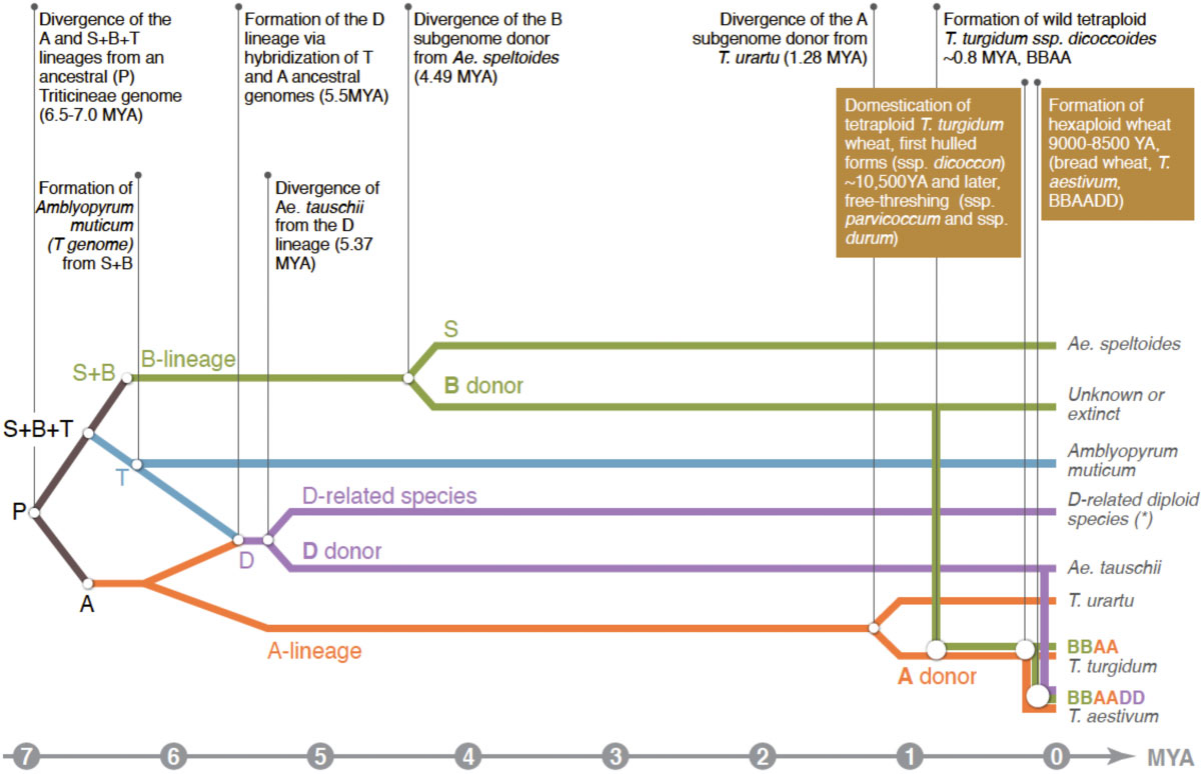
Phylogenetic representation of wheat evolution. Wheat evolution is shown starting ∼7 MYA from a progenitor that gave rise to the (A), (B), and (D) lineages that merged to form bread wheat. The relative timing of the major speciation events is shown in the horizontal axis and described in the boxes above. The tree is adapted from Glémin et al. (2019); Li et al. (2022); Avni et al. (2021). The diploid species related to the D genome (asterisks) are *Aegilops bicornis*, *Ae. longissima*, *Ae. searsii*, *Ae. sharonensis*, *Ae. caudata, Ae. comosa, Ae. umbellulata*, and *Ae. uniaristata*.

A major milestone in the study of wheat evolution was the discovery of the chromosome number of the wheat species (Sakamura, 1918; Sax, 1918), showing that the wheats comprise a polyploid series consisting of diploids (2*n* = 2*x* = 14), tetraploids (2*n* = 4*x* = 28), and hexaploids including bread wheat (2*n* = 6*x* = 42). Soon after this discovery, Kihara (1919, 1924), Sax (1921, 1922), and others (see tribute to Elisabeth Schiemann [Kilian et al., 2014; and references therein]) crossed representatives of the different ploidy levels and found that polyploid wheats are allopolyploids, that is, having subgenomes (chromosome sets) that derived from different species. When hexaploid bread wheat was crossed with domesticated emmer tetraploid wheat, *T. turgidum* ssp*. dicoccon* (genome BBAA 2*n* = 4*x* = 28), analysis of chromosomal pairing at meiosis of the F_1_ pentaploid hybrids indicated that hexaploid wheat, *T.* *aestivum*, contains the B and A subgenomes of tetraploid wheat with an additional diploid subgenome (Kihara, 1919; McFadden and Sears, 1944, 1946). Morphological (Kihara, 1944) and cytogenetic studies (McFadden and Sears, 1944, 1946) showed that the third subgenome of hexaploid wheat, designated D, was derived from *Aegilops* *tauschii.*  McFadden and Sears (1946) produced a synthetic hexaploid from the cross of tetraploid emmer wheat with *Ae. tauschii,* that resembled the hulled form of *T. aestivum*, ssp. *spelta* in most morphological traits, had the hexaploid number of chromosomes, and produced fertile F_1_ hybrids with *T. aestivum* exhibiting complete chromosomal pairing at meiosis. This demonstrated unequivocally that *Ae. tauschii* (formerly *Ae. squarrosa*), is the donor of the third subgenome of *T. aestivum.* When the genome of *Ae. tauschii* was sequenced, first as a draft (Jia et al., 2013), then as a high-quality sequence (Luo et al., 2017; Zhao et al., 2017), it became apparent that the origin of the D genome, derived 5.37 million years ago (MYA) from the D-lineage, resulted from hybridization ∼5.5 MYA (Figure 1) between the ancestral donors of the A and B subgenomes (Marcussen et al., 2014).

The wild progenitor of tetraploid wheat that contributed to the A and B subgenomes of *T. aestivum* had been discovered in nature in 1906 by Aaronson (Aaronsohn, 1910). The fertile F_1_ hybrids between ssp. *durum,* ssp*. Dicoccon*, and wild emmer wheat (*T. turgidum* ssp*. dicoccoides*) (Tschermak, 1914), as well as the subsequent demonstration of the full pairing of their chromosomes (Sax, 1921, 1922; Percival, 1921; Kihara, 1924) indicated that wild emmer was the wild progenitor of domesticated tetraploid wheat (Figure 1) and thus of the A and B subgenomes of *T. aestivum*. Whole-genome sequencing confirmed the high synteny between ssp*. dicoccoides* (Avni et al., 2017) and ssp*. durum* (Maccaferri et al., 2019).

Thus, bread wheat evolved through two rounds of allopolyploidization (Figure 1). The first event led to the formation of tetraploid wild emmer wheat approximately 800,000 years ago following hybridization between two diploid species: the male donor of the A subgenome, a species very similar to *Triticum* *urartu*, but that diverged from it ∼1.3 MYA, and the female donor of the B subgenome, a species related to *Ae.* *speltoides*, but that diverged from it ∼4.5 MYA (Marcussen et al., 2014; Li et al., 2022). The second allopolyploidization event that produced hexaploid wheat involved allotetraploid wheat as the female donor of the B and A subgenomes and *Ae. tauschii* as the male donor of the D subgenome. This event first formed hulled allohexaploid wheat from which the more advanced free-threshing forms originated by mutations (Feldman, 2001). The cytoplasm of *T. aestivum*, designated B, is identical to that of its maternal parent, *T. turgidum* (Tsunewaki, 2009, and references therein).

While the identity of the diploid donors of two out of the three subgenomes of *T*. *aestivum*, namely, *T. urartu* (A subgenome) and *Ae. tauschii* (D subgenome), is well established, that of the B subgenome, the cytoplasm donor of bread wheat, remains elusive. There is no extant diploid species whose chromosomes have a high affinity for the B subgenome, suggesting that the progenitor is either extinct or remains to be discovered, or that the B subgenome has a polyphyletic origin and evolved at the polyploid level through multiple hybridizations with close extant species, for example, the five species from the Sitopsis section: *Ae. speltoides*; *Ae. bicornis*; *Ae. sharonensis*; *Ae. Longissima*; and *Ae. searsii.* Recent insight from whole-genome sequences suggests that *Ae. speltoides* diverged from the B subgenome ∼4.5 MYA (Li et al., 2022). The estimate that wild emmer wheat (genome BBAA) was formed ∼0.8 MYA (Marcussen et al., 2014) rules out the possibility that *Ae. speltoides* is the direct progenitor of the B subgenome. The results of Li et al. (2022) support the hypothesis that the donor of the bread wheat B subgenome is a diploid species closely related to, but distinct from extant *Ae. speltoides.* Moreover, despite evidence for introgressions from other Sitopsis species, the B subgenome appears to have a mainly monophyletic origin. According to this scenario, the B subgenome donor is either extinct or remains to be discovered. Considering that morphological differences are sometimes very subtle among Sitopsis species, it cannot be ruled out that the B subgenome donor is still present in the wild. The B subgenome donor and *Ae. speltoides* thus belong to the B lineage, and both diverged from *Amblyopyrum muticum* (2*n* = 2*x* = 14, T genome) ∼6.4 MYA (Figure1), making *Am. muticum* the most ancestral extant representative of the B lineage (Glémin et al., 2019; Bernhardt et al., 2020; Avni et al., 2021; Li et al., 2022).

In summary, one could say that bread wheat was formed from its diploid and tetraploid progenitors through a cycle of “diverge-and-merge” speciation events. Indeed, after their divergence ∼7 MYA, the A and B genomes merged to form a new homoploid hybrid species, *Ae. tauschii* (genome DD) 5.5 MYA, then, 800,000 YA, A and B reunited again, but this time to form an allotetraploid species (ssp*. dicoccoides,* genome BBAA). And to close the cycle of “diverge-and-merge,” the D genome, which is itself an ancient homoploid hybrid between B and A, merged with the BBAA genome approximately 9,000 YA to form allohexaploid bread wheat (Figure 1). Another insight from recent studies points to the seminal role of *A.* *muticum* as an ancestral contributor to both the B and D lineages (Glémin et al., 2019; Avni et al., 2021; Li et al., 2022).

## Evolution under allopolyploidy

### Speciation

Allopolyploidization is a biological process that played a central role in plant speciation and evolution (Soltis and Soltis, 2009), including in wheat, which is an archetype of evolution via polyploidization (Feldman and Levy, 2012). It constitutes a radical and rapid mode of speciation that produces a new species by means of inter-specific or inter-generic hybridization of two diverging species, followed by chromosome doubling of the F_1_ hybrids. There has been much debate on the fitness of polyploids (Stebbins, 1950, 1971; Soltis and Soltis, 1999; Mayrose et al., 2011). A recent meta-analysis of tens of thousands of species (Rice et al., 2019) suggests that the prominence of polyploidy depends on several factors, for example, polyploids are relatively more abundant in cold or dry climates with less species richness, presumably thanks to facilitated competition with endemic diploids. Such ability to outcompete diploid relatives can be achieved through a diversity of mechanisms, including novel intergenomic interaction, rewiring of gene expression, new dosage effects, new protein complexes, and fixed heterosis (Birchler and Veitia, 2021). Domestication, a recent event in plant evolution, seems to have favored allopolyploids in wheat and in several other crop species, possibly as it allowed for rapid adaptation to the new cultivation environment (Dubcovsky and Dvorak, 2007). Diploid einkorn wheat is grown on a smaller scale than tetraploid durum, which in turn represents only ∼10% of the area and production of hexaploid bread wheat.

All diploid grass species are thought to be paleopolyploids that underwent at least one event of whole-genome duplication (Salse et al., 2008; Wang et al., 2015; Murat et al., 2017), and on the evolutionary scale, polyploidization was followed by cytological and genetic diploidization (Bolot et al., 2009). Genetic diploidization can be considered as proposed by Ohno (1970), as a regulatory process that brings redundant or unbalanced gene systems in allopolyploids toward a diploid-like mode of expression. This can be achieved through multiple mechanisms (Conant et al., 2014), such as gene mutation, elimination, pseudogenization, or gene dosage compensation (Galili et al., 1986). Likewise, cytological diploidization is essential for the success of the new allopolyploid species to achieve full fertility and to provide a stable disomic inheritance for new beneficial interactions between subgenomes (Feldman and Levy, 2012).

### Karyotypic evolution

The ancestral Triticeae karyotype has been proposed to have derived from the *n* = 12 ancestral grass karyotype as seen in rice (Salse et al., 2008; Luo et al., 2009). Comparative genetics showed that the reduction from *n* = 12 to *n* = 7, without the loss of genes, was accomplished through four centromeric ancestral chromosome fusions (leading to functional monocentric neochromosomes), one fission, and two telomeric ancestral chromosome fusions (Salse et al., 2008; Luo et al., 2009). The Triticeae basic chromosome number of *n* = 7 evolved via the loss of five functional centromeres, four of which correspond to those of rice chromosomes Os4, Os5, Os6, and Os9, and the fifth to either that of Os3 or Os11 (Luo et al., 2009).

The genome of common wheat, *T.* *aestivum,* shares the ancestral reciprocal translocation (between A4 and A5 on the A subgenome) as well as an additional lineage-specific translocation (between chromosomes 4A and 7B) to reach the modern 21 chromosomes (Salse et al., 2008; Luo et al., 2009; Murat et al., 2014). One of the most prominent large-scale karyotypic variants that occurred in wheat is a translocation between chromosomes 5B and 7B. This translocation was found in the majority of the lines tested by Walkowiak et al. (2020). Sequence data showed that the translocation between chromosomes 5B and 7B, which are ∼737 and 762 Mb long, respectively, gave rise to translocated chromosomes of 488 Mb (5BS/7BS) and 993 Mb (7BL/5BL) in length. The translocation breakpoint was mapped to a ∼5-kb GAA microsatellite when comparing cv. ArinaLrFor and SY Mattis.

In addition to the series of rearrangements that shaped the present-day bread wheat chromosomes, functional units, such as centromeres or nucleolar organizers, have also rapidly evolved under allopolyploidy in wheat. The Triticeae centromeres are no exception to other plants, forming through the assembly of the CENH3 proteins with arrays of satellite repeats and retroelements (Cheng and Murata, 2003). The primary constrictions of barley, wheat, *Aegilops*, and rye chromosomes, that is, the physical locations of centromeres, harbor retroelement-like sequences (Presting et al., 1998; Fukui et al., 2001; Cheng and Murata, 2003). In wheat, a detailed chromatin immunoprecipitation analysis, using a CENH3 antibody followed by sequencing of the precipitated DNA fraction, enabled the identification of a distinct dynamic structure for wheat centromeres, possibly due to evolution through frequent hybridization and polyploidization (Su et al., 2019). Unlike typical plant centromeres, which carry a satellite of tandem repeats ranging from 150 to 180 bp, the wheat CENH3 nucleosomes were associated with two different types of repeats much larger than other plant centromeric repeats, namely 550 and 566 bp long, respectively (Su et al., 2019). Moreover, different subgenomes tended to contain different repeat types, and some chromosomes lacked satellite repeats altogether. Phylogenetic analyses indicate that the repeat signals were stronger in diploids than in polyploids and that centromere structure evolved rapidly at the polyploid level (Su et al., 2019).

The nucleolar organizing regions (NORs) where nucleoli form is seen as secondary constrictions on chromosomes (McClintock, 1934). NORs contain ribosomal (rDNA) genes that code for the ribosomal RNA (rRNA) and are organized in a gene array of thousands of tandemly arranged copies per NOR. The total number of rDNA units in the fully sequenced genome of cv. Chinese Spring was estimated at 11,160 copies corresponding to 100 Mb, 30.5% of which are on the Nor-B1 locus (Chr. 1B), 60.9% on Nor-B2 (Chr. 6B), and 8.6% in other NORs (Handa et al., 2018), which is consistent with earlier estimates (Flavell and O’Dell, 1979; Appels and Honeycutt, 1986). In hybrids and polyploids, the rDNA genes of one parental set are transcribed, while most or all rDNA genes inherited from the other parent remain silent. This phenomenon, known as nucleolar dominance (Pikaard, 2000), is common in the allopolyploid species of the genera *Aegilops* and *Triticum* (Feldman et al., 2012). The diploid species of wheat, *Triticum* *monococcum* and *T. urartu*, contain two nucleolar organizer regions, one on chromosome arm 1AS and the second on 5AS (Miller et al., 1983). In the allopolyploid wheat species, the NOR of 1AS is inactive, while that of 5AS was lost (Miller et al., 1983; Jiang and Gill, 1994). Newly synthesized allopolyploids exhibit genetic and epigenetic changes in their rRNA-encoding genes similar to those occurring in natural allopolyploids, indicating that these changes are rapid and reproducible and were generated early during allopolyploid formation (Baum and Feldman, 2010). Guo and Han (2014) conducted a detailed molecular analysis of the fate of wheat NORs in several allopolyploids, including the synthetic and natural BBAADD genomes. They concluded that the NORs from the B subgenome are dominant in several genomic combinations and that the elimination of the other NORs proceeds in two steps—first through silencing and hypermethylation in the first generations, then through progressive elimination of the non-B rDNA copies, starting in the fourth and ending by the seventh generation after polyploidization (Guo and Han, 2014).

### Evolution of gene order and content

Allopolyploidy involves genetic redundancy, even in an allohexaploid where the different subgenomes diverged from each other millions of years ago. This was shown in a milestone study by Ernie Sears with the demonstration that each of the seven pairs of chromosomes of a subgenome could compensate for the absence of a specific pair of another subgenome (Sears, 1954, 1959). The compensating pair was called homoeologous (with partially homology). The wheat genome could thus be arranged into seven homoeologous groups and three subgenomes (Sears, 1959). From an evolutionary point of view, this study also implied that, despite their divergence, the subgenomes maintained a relatively high degree of similarity.

Pioneering molecular studies revealed a high level of gene synteny and collinearity in the homoeologous chromosomes of bread wheat (Moore et al., 1995). The nature of the divergence/similarity between the hexaploid wheat subgenomes was fully understood only after the publication of an annotated high-quality sequence of bread wheat cv. Chinese Spring, the same cultivar used by Ernie Sears in his analysis of wheat chromosomes homoeology (IWGSC, 2018). Annotation of *CS* genes identified 107,891 “high confidence” genes distributed almost equally between the A, B, and D subgenomes. Synteny was highly conserved in the three subgenomes. Synteny was more prominent, with larger syntenic blocks, in interstitial regions compared to sub-telomeric regions or pericentric regions (IWGS, 2018). As shown by Akhunov et al. (2003), a higher rate of gene duplication and deletion in the sub-telomeric regions was in part responsible for the reduction in synteny. Only 55% of the genes had a syntenic homoeolog in all three subgenomes, whereas 15% had at least one missing homoeolog but had a paralog. The percent of missing homoeologs (gene loss) was similar (∼10%) in each subgenome (IWGS, 2018).

Gene organization within the chromosome analyzed through sequencing of single wheat BAC clones and BAC clone contigs suggested that genes are clustered into many small islands of 3–4 genes interspersed by retroelements (Feuillet and Keller, 1999; Wicker et al. 2005; Choulet et al. 2010). Gene density in the distal regions of chromosome 3B of common wheat was higher than in the rest of the genome due to a higher incidence of duplicated genes in these regions (Dvorak and Akhunov, 2005; Luo et al. 2013). For example, when comparing the *Ae. tauschii* and bread wheat D subgenome, only 87.4% of the genes were present at the expected orthologous position, highlighting a dynamic gene copy-number variation that is not related to whole-genome duplication (Zhou et al., 2021). Some gene families, such as prolamine genes (including glutenins and gliadins) and disease-resistance genes were among those most prone to be nonorthologous with other grasses (Zhou et al., 2021).

In bread wheat, gene family expansion generally occurred in the wild progenitor or the common ancestor of the subgenomes: out of 8,592 expanded families, 6,216 expanded in all three A, B, and D subgenomes while 1,109 expanded in only one of the subgenomes, and only 78 gene families contracted (IWGS, 2018). Interestingly, there were significant differences between subgenomes when functions were assigned to the families that expanded: there was an over-representation of seed-related genes (embryo and endosperm) in the A subgenome and of vegetative growth and development in the B subgenome. Families that expanded in all three genomes were enriched in genes that were hypothesized to play an important role in wheat breeding for trait related to yield or biotic and abiotic stress resistance (IWGS, 2018); however, a causal relationship has not been shown to date.

Walkowiak et al. (2020) analyzed the genomic sequence of different bread wheat lines and found high collinearity of gene order on homologous chromosomes and overall similar genome sizes. Nevertheless, ∼12% of the genes showed a “Presence/Absence Variation” between the different cultivars. Deletions in the polyploid background can be 10 times faster than in the diploid background (Dvorak and Akhunov, 2005). On the other hand, the repeat-rich intergenic environment of genes can accelerate gene duplication by 20-fold (Akhunov et al., 2007). Indeed, repeats, often TEs, can promote gene duplication by unequal (Molinier et al., 2004) or ectopic crossover (Shalev and Levy, 1997), or by gene-flanking helitrons (Morgante et al., 2005), or by capturing genes in mobile structures (Jiang et al., 2004). This dynamic gene copy number variation might be made possible by the buffering effect of whole-genome duplication enabling toleration of a broad range of genetic changes over time.

### Rapid genetic and genomic changes leading to genetic and cytological diploidization

Genome merging in hybrids or allopolyploids generates both cytological and genetic challenges to the nascent species. For example, homoeologous pairing may lead to partial sterility and multisomic inheritance, and genomic clashes between regulatory elements of different subgenomes can lead to hybrid necrosis (Bomblies and Weigel, 2007). Rewiring of gene expression (Tirosh et al., 2009) may also reduce fitness of the nascent species. Presumably, a new allopolyploid species unable to rapidly overcome such challenges would not be able to establish itself and survive in nature.

Upon allopolyploidization, the immediate triggering of a variety of cardinal genetic and epigenetic changes that affect genome structure and gene expression might be essential to bring about genetic and cytological diploidization. Such rapid changes have been reported in newly formed allopolyploids, including elimination of coding and noncoding DNA sequences, transposable element (TE) and tandem repeat elimination or amplification, and gene expression modifications (Feldman et al., 1997; Liu et al., 1998; Ozkan et al., 2001; Shaked et al., 2001; Han et al., 2003, 2005; Salina et al., 2004; Ma and Gustafson, 2006; Baum and Feldman, 2010; Guo and Han, 2014). Remarkably, sequence elimination is often reproducible, as is evident from the elimination of the same sequences in synthetic and natural allopolyploids bearing the same genomic combinations (Feldman et al., 1997; Ozkan et al., 2001; Han et al., 2005). In many instances, sequence elimination leaves homologous-specific sequences in the genome, instead of two or three originally present in the parental genomes. Such differential elimination leads to rapid divergence of the homoeologous chromosomes, contributing a physical basis to facilitate chromosome homology recognition, and enabling hexaploid wheat to exhibit exclusively intra-subgenomic pairing in the form of bivalents between fully homologous chromosomes (diploid-like meiotic behavior). Allopolyploid species of the wheat group contain 2%–10% less DNA than the additive sum of their parental species, and synthetic allopolyploids exhibit a similar loss, indicating again that DNA elimination occurs soon after allopolyploidization (Furuta et al., 1974; Eilam et al., 2008).

TEs activity can be triggered by allopolyploidization, affecting gene expression and inducing many structural rearrangements, including deletions or duplications (Kashkush et al., 2002, 2003; Yaakov and Kashkush, 2011; Senerchia et al., 2015; Bariah et al., 2020). Due to their high copy number, TEs have the highest potential to differentiate between subgenomes. In bread wheat, TEs constitute ∼80%–85% of the total genome with ∼70% long terminal-repeat retrotransposons (LTRs) and ∼12% DNA transposons (Walkowiak et al., 2020). LTR elements that are “young,” that is, that have transposed recently, tend to be located in the gene-rich recombinogenic distal part of chromosomal arms, while “old” LTR elements tend to be conserved among wheat lines and to be located in the pericentric heterochromatic regions of the chromosomes (Walkowiak et al., 2020). Recent transpositions, therefore, contribute to subgenome divergence in regions important for promoting homologous pairing. TEs also have the potential to affect genome structure and function through transposition, induction of homologous recombination, and epigenetic re-patterning (Shalev and Levy, 1997; Bennetzen, 2005; Slotkin and Martienssen, 2007; Fedoroff, 2012). Since the activity of TEs is governed by epigenetic regulation, their activation might be induced by genetic and environmental perturbations (Fedoroff, 2012). Hence, TEs may mutate genes, alter gene regulation, and generate new genes, in response to environmental challenges, thus providing fuel for evolution (Kidwell and Lisch, 1997). TEs associated with genes can affect the transcription of neighboring genes, as shown for retroelements, through readout activity of their LTRs (Kashkush et al., 2002, 2003). Interestingly, inspection of the expression of homoeologs for which all three subgenomes were represented (triads) showed that for ∼70% of the triads, the level of expression was balanced, that is, homoeoalleles were generally expressed at the same level. For the ∼30% remaining triads, differential expression was often associated with the diversity of TEs in proximity to the promoter region (Ramírez-González et al., 2018). Wheat TEs can affect splicing and intron retention as shown for SINEs that are enriched in introns (Keidar et al., 2018). Miniature inverted-repeat transposable elements (MITEs) also show a strong association with wheat genes, being near genes, or within the transcriptome (Keidar-Friedman et al., 2018). All this suggests that TEs are drivers of dynamic genomic changes and modulation of gene expression and contribute massively toward shaping genome structure, gene expression, and evolution. However, these dynamic changes, which can lead to deleterious genome instability, are mitigated by several epigenetic genome stabilizing factors that suppress TE activities such as cytosine methylation (Fedoroff, 2012) or small RNAs (Kenan-Eichler et al., 2011). Nevertheless, TEs have retained some extent of mobility throughout wheat evolution as suggested from their high diversity between the subgenomes of bread wheat and between varieties in the same subgenome (Wicker et al., 2018) and as shown for MITEs in a newly synthesized wheat allohexaploid (Yaakov and Kashkush, 2012).

Introgression was an important mechanism to increase genetic diversity during wheat evolution. Indeed, there has been extensive natural introgression from wild emmer wheat into the background of domesticated emmer wheat or bread wheat, leading to higher variability in these genomes (He et al., 2019; Zhou et al., 2020). This was facilitated by the ease of crossing between wild emmer and domesticated subspecies of *T. turgidum*, having the same BBAA genome. Moreover, the initial process of domestication might have taken approximately 2–3,000 years (Kislev, 1984; Tanno and Willcox, 2006) as seen from the archeological records where fragile, partly fragile, and nonfragile spikes can be found in the same farming sites, providing high chances to hybridize. Consistent with these biological and archeological data, the variation in the domesticated-emmer genome includes ∼73% of that of wild emmer (Zhou et al., 2020; Keilwagen et al., 2022; Sharma et al., 2021). Genotyping of *T. aestivum* landraces has shown that a significant part of the variation in wild or domesticated tetraploid wheat has also found its way into the A and B subgenomes of the bread wheat genome suggesting that there was only a limited genetic barrier between tetraploid and hexaploid wheat (Dvorak et al., 2006). The barrier between diploid *Ae. tauschii* and hexaploid wheat is harder to cross because the resulting genome ABDD tetraploid hybrid is highly sterile, which is consistent with the finding that the variability of the D subgenome is 2–5 times lower than that of the A and B subgenomes (Caldwell et al., 2004). Nevertheless, some degree of introgression took place as seen from whole-genome sequencing (Gaurav et al., 2021; Zhou et al., 2021). Introgressions were found between nonhomologous genomes, seen as an incongruent block of single-nucleotide polymorphisms (SNPs) that are specific to another species and do not follow the phylogeny of the species, mostly from the B to D lineage and to a lesser extent from D to B lineage. However, these introgressions were relatively rare and took place prior to the formation of tetraploid wheat (Li et al., 2022).

### Homologous and homoeologous recombination

Meiotic recombination between homologous chromosomes is a major engine of diversity in wheat evolution and breeding. Introgressions via recombination between homologous genomes of bread wheat and wild relatives have been relatively frequent for the A and B genomes as discussed above. Dvorak (2009) reviewed data indicating that Triticeae genomes have a steep recombination gradient along the centromere–telomere axis following gene content which increases from the proximal toward the distal region. In their detailed study of the pattern of crossovers along the sequenced chromosome 3B of bread wheat, Saintenac et al. (2009) also show that crossover frequency increases gradually from the centromeres to the telomeres. Moreover, they show that small chromosome segments of high gene density and frequent recombination interspersed with relatively large regions of low gene density and infrequent recombination (Saintenac et al., 2009). Multiple crossovers occurred within these gene-dense regions where the degree of recombination is at least 11-fold greater than the genomic average (Faris et al., 2000). Fine mapping of crossover in chromosome 3B showed that 82% of the crossovers occurred in 19% of the chromosome length in the sub-telomeric regions which carry 60%–70% of the genes (Valenzuela et al., 2013; Darrier et al., 2017). The remaining ∼35% of the genes are located in recombinationally poor chromosomal regions (Erayman et al., 2004; Darrier et al., 2017), hindering the elimination of deleterious mutations or introgression of beneficial ones in these regions. Recombination tends to occur in or near genes, often in promoter regions (Darrier et al., 2017). Most recombination events take place in hotspots that are characterized by specific sequence motifs, such as CCN or CTT repeats, or A-rich regions (Darrier et al., 2017) and are associated with typical chromatin modifications (Liu et al., 2021). A new member of the *RecQ* helicase gene family was recently shown to be associated with high crossover frequency in hexaploid wheat (Gardiner et al., 2019).

Superimposed on the divergence of the homoeologous chromosomes is a genetic system that contributes to the maintenance and reinforcement of the exclusive bivalent pairing in the allopolyploid *Triticum* species, ensuring that chromosome pairing is wholly restricted to homologous chromosomes (reviewed by Sears, 1976). The most potent suppressor of homoeologous pairing between wheat subgenomes and in interspecific or intergeneric hybrids is a gene designated *Pairing homoeologues1* (*Ph1*). First described by Okamoto (1957), Sears and Okamoto (1958), and Riley and Chapman (1958), *Ph1* is located on chromosome arm 5BL and suppresses homoeologous chromosome pairing without affecting homologous chromosome pairing. A deletion of *Ph1* was induced by Sears (1977) via X-irradiation and shown by Gill and Gill (1991) to encompass 73.5 Mbp (the *ph1b* locus). In the absence of *Ph1*, pairing occurs between the homoeologues of the A, B, and D subgenomes of bread wheat or between homoeologous chromosomes in hybrids between hexaploid wheat and wild relatives (Sears, 1976; Feldman, 1988). Later analysis of deletions in 5B enabled restricting the size of the *Ph1* locus to a 2.5-Mb region (Griffiths et al., 2006). This region still contains several candidate genes that may affect chromosome synapsis and recombination: *C-Ph1* (Bhullar et al., 2014), *CDK2-like* genes, methyl transferase genes and *Hyp3*, now called *TaZIP4-B2*, and it is possible that the *Ph1* phenotypes are due to the combined effect of several genes as proposed by Griffiths et al. (2006). Recently, two mutants in *TaZIP4-B2*, a point mutation induced by ethyl methanesulfonate (EMS) and a small deletion induced by clustered regularly interspaced short palindromic repeats (CRISPR)–Cas9 yielded high levels of homoeologous pairing (Rey et al., 2017) making it a strong *Ph1* candidate. Nevertheless, recovering the whole complexity of the *Ph1* phenotype might require mutations in additional genes at the same locus. In addition to *Ph1*, there are at least two other suppressors of homoeologous pairing in *T. aestivum*, the *3DS* and *3AS* genes, designated *Ph2* and *Ph3,* respectively, that are less potent than *Ph1* (Sears, 1982, 1984). The 3DS *Ph2* locus was recently isolated (Serra et al., 2021) and found to encode the wheat homolog of DNA mismatch repair gene *MSH7*, *TaMSH7*.

How homoeologous suppression evolved is still poorly understood. It seems to have existed at the diploid levels (Attia et al., 1979). However, diploid activity seems to have been too weak to explain homoeologous suppression as seen in bread wheat. Riley (1960) and Sears (1976) assumed that *Ph1* evolved at the allotetraploid level, in parallel to, or soon after, the allopolyploidization process. The evolution of *Ph1* seems to have been gradual with activity increasing with polyploidy with diploid < tetraploid < hexaploid (Ozkan and Feldman, 2001). Tracing the evolution of homoeologous pairing suppression will be possible only when the molecular mode of action of *Ph1* is more fully understood but will remain limited in the absence of an extant B donor species.

In nature, the number of introgressions that took place by homoeologous recombination in the bread wheat lineage seems to have been relatively infrequent (Li et al., 2022). Nevertheless, when it happens, as shown in a synthetic allotetraploid lacking *Ph1*, homoeologous recombination events were shown to occur preferentially within exons, generating novel hybrid transcripts and proteins (Zhang et al., 2020a), possibly due to the higher homology in these regions. This contrasts with homologous recombination that preferentially occurs in promoters (Darrier et al., 2017).

In summary, allopolyploidy in the wheat genome has enabled, and in some cases induced, rapid changes at many levels, starting from providing a rapid speciation mode that triggered massive changes in repetitive DNA such as centromeres, NORs, and TEs, as well as gene elimination and duplication, thus leading to divergence of homoeologous genomes. Despite these rapid alterations, stability has been maintained thanks to epigenetic regulation and to a genetic system superimposed on the diverged homoeologs, namely the *Ph1* locus, that was present in diploids but further evolved in polyploids, controlling the preferential pairing of homologous rather than homoeologous chromosomes. It is assumed that all these mechanisms have contributed to the rapid genetic and cytological diploidization of the wheat genome, ensuring disomic inheritance and fertility of the nascent species, eliminating redundancies, and maintaining heterotic interactions between subgenomes.

## Wheat domestication

**Figure 2:**
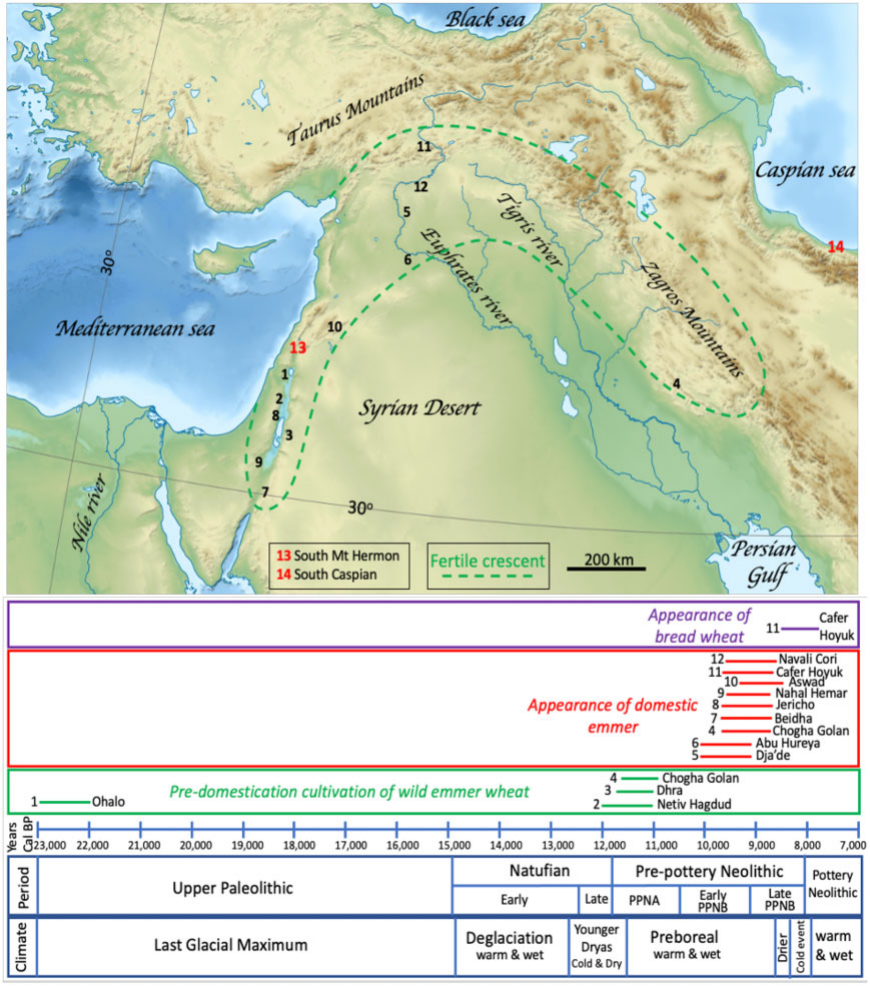
Archaeological evidence for wheat cultivation and domestication in the near-east. The location of the fertile crescent is shown as dashed green lines. Its boundaries correspond to the distribution of wild progenitors of wheat, barley, and several legumes as well as to early domestication of these crops. The western part, called the Levant or levantine corridor (Bar-Yosef, 1998), goes south, around the Jordan valley between the dashed line on the side of the Syrian desert and the Mediterranean Sea. The south Levant is the region between Beidha (#6) and Aswad (#9) and the north Levant is north of Aswad (#10), for example, in Dja’de (#5) and Abu Hureya (#6). The northern area of the fertile crescent is also referred to as the upper Euphrates (e.g. Cafer Hoyuk, #11), and the east of the fertile crescent, in the Zagros mountain is represented by sites such as Chogha Golan (#4). The years on the blue horizontal axis correspond to “Calibrated years before present” (Cal BP). The bottom boxes represent the climatic and the archeological periods when cultivation and domestication took place. Horizontal lines flanked by the location number (see map) and names indicate the relevant period when archeological evidence of cultivation or domestication was found. Numbers in red indicate two regions where evidence came from genomic data rather than archaeological data for the putative progenitors of domestic emmer (#13) and for the donor of the D subgenome of bread wheat (#14). Archaeological data were gathered from Nesbitt (2001); Willcox (2012); Zeder (2011); Riehl, et al. (2013). A blank topographic map from Wikipedia (Middle East topographic map-blank 3000bc.svg, by Fulvio314, CCBY 3.0) served as the background on which text and data were added.

Wheat is a staple food of humanity and its evolution under human selection is part of a never-ending endeavor to reach food security. Proto-humans likely fed on foraged wheat since the Paleolithic. What induced hunter–gathers in the Near East to start cultivation and then to domesticate plants and become farmers? This question has inspired myths and religions (e.g. Genesis 3: 17–23) and has fascinated archeologists, anthropologists, plant geneticists, and evolutionary biologists, including Darwin (1868). Despite various hypotheses, such as pressure on food resources due to an increase in human population and to climate change (see Figure 2), the “why” of wheat cultivation and domestication remains a mystery. Likewise, an important and unanswered question is whether domestication was an intentional process, namely due to active selection by early farmers (as modern breeders would do) of mutants adapted to cultivation, such as nonfragile spike and free threshing, or whether it was a genetic drift due to the increased fitness under cultivation of these mutants that became fixed after several generations. Both are plausible and not mutually exclusive scenarios.

### Wheat domestication: where and when?

The phases of wheat evolution under human selection are: (1) the gatherers (also called foraging) period when wheat was harvested from wild stands; (2) a predomestication cultivation period when wild wheat was grown in small plots; (3) the domestication of wild emmer wheat, namely, the period when mutations in genes affecting spike morphology appeared and became fixed. These mutations, which hindered seed dispersal and facilitated threshing, suited the farmer rather than the wild plant and turned wheat into an organism dependent on humans for its dissemination; (4) the formation of bread wheat under domestication; (5) the spread of bread wheat and the accumulation of landraces; and (6) the green revolution and modern breeding.

We know little of the predomestication period, when human were gatherers and brought grains near their dwellings, due to a limited amount of available data. The earliest data relevant to wheat comes from archeological records from the upper paleolithic Last Glacial Maximum period approximately 23,000 years ago in the hunter–gatherer sedentary camp of Ohalo II on the shores of the lake of Galilee (Figure 2), suggesting that there might have been a period of cultivation of wheat that was not yet domesticated (Snir et al., 2015). The evidence is that extensive farming-related activity was detected at this site, as deduced from various flints, sickle blades and stone grinding tools, fauna remains, and large amounts of seeds (approximately 10,000 seeds from cereals) including wild emmer wheat, wild barley, and wild oats, in what was a human-disturbed environment containing seeds from weedy species (presumably a field). If this interpretation is correct, this would be the earliest known site of wild wheat cultivation. Alternatively, it might be a site where wild emmer wheat, harvested from nearby wheat stands, was brought and processed (Piperno et al., 2004). Ohalo II is a singular case as there is a gap of approximately 10,000 years before other human sedentary settlements were found during the Younger Dryas in the Late Natufian and early Pre-pottery Neolithic-A period throughout the fertile crescent (Figure 2). There is a consensus among archaeobotanists (not considering Ohalo II) that the cultivation of wild cereals predated morphological domestication by more than 1,000 years with wild einkorn in the north Levant as soon as 13,000 Cal-Y BP (Abu Hureyat I and Mureybit I-III) and 11–12,000 Cal-Y BP, wild emmer in the south Levant in Netiv Hagdud and Zaharat adh-Dhra (see reviews by Nesbitt, 2001 and Willcox, 2012) and wild barley and to a lesser extent also wild emmer in the eastern part of the fertile crescent Chogha Golan site in the Zagros mountains (Riehl et al., 2013). During these periods, there was no clear morphological evidence for the presence of domesticated wheat.

**Figure 3:**
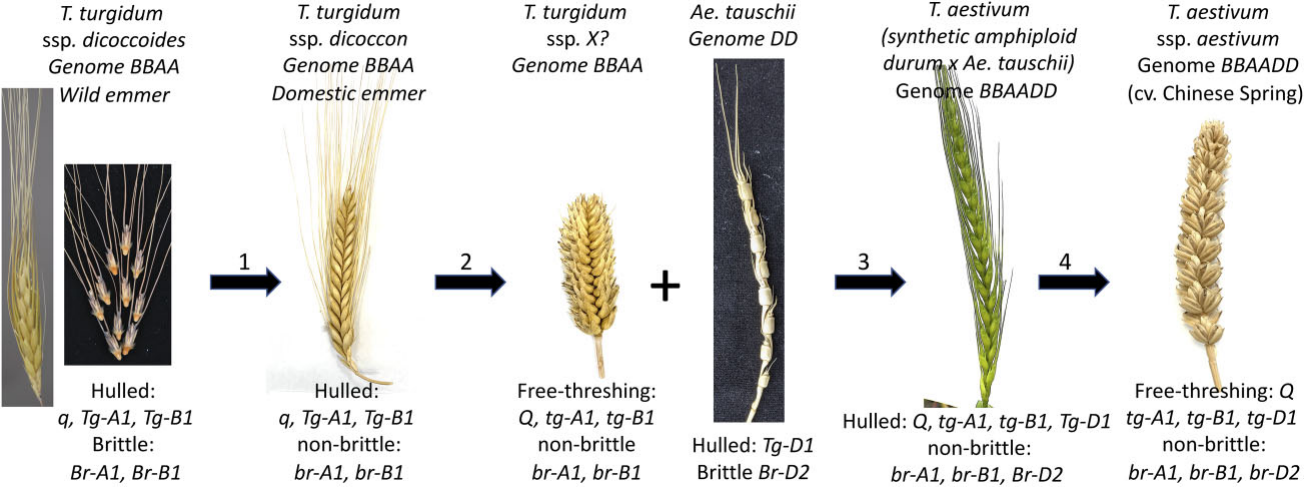
Major mutations and morphological changes during wheat domestication. 1, The transition from ssp*. dicoccoides* to ssp*. dicoccon* involved mutation in the *Brittle rachis* loci. Some modern emmer wheat lines might also contain mutations in some but not all loci affecting free threshing. 2, Free-threshing tetraploid wheat, named ssp*. parvicoccum*, appears in the archeological record approximately 2,000 years before *ssp. durum*. It is now extinct but might have resembled the tetraploid wheat (Genome BBAA) shown here as ssp. X that was extracted from hexaploid wheat and has a compact spike and small grains. Its genotype must have been similar to durum, namely free threshing with soft glumes, with mutants Q and *tg-A1, tg-B1.* 3, The hybridization of this free-threshing tetraploid wheat with the DD subgenome donor, *Ae. tauschii*, gave rise to a primitive hulled hexaploid wheat, different from spelt wheat due to the Q factor, and absent from the archaeological record. It likely resembled the picture shown from a synthetic hexaploid between ssp*. durum* and *Ae. tauschii* shown here. 4, Soon after its formation, hexaploid wheat became free threshing thanks to a mutation in *Tg-D1* and its rachis became thicker thanks to a mutation in *Br-D2*.

The morphological hallmark of domesticated cereals is the nonbrittle rachis, marking the transition from seed dispersal through spike fragility to dispersal by farmers (Figure 3). Domesticated emmer wheat appears first in the archeological record of the northern Levant (Figure 2) at Dja’de and Abu Hureya II in the early PPNB (starting approximately 10,000 Cal-Y BP), and briefly after that in the southern Levant at Beidha, Jericho, Nahal Hemar, in the central fertile crescent at Cafer Hoyuk and Navali Cori (reviewed by Nesbitt, 2001 and Willcox, 2012) and east of the fertile crescent at Chogha Golan (Riehl et al., 2013). Early genetic studies searching for the location of emmer wheat domestication did not provide clear answers and did not always match archaeological data, with evidence pointing to either the upper Euphrates or the Levant (Ozkan et al., 2005; Luo et al., 2007; Civan et al., 2013). Confusion on these points was exacerbated by the mobility of Neolithic farmers, rapidly spreading seeds and cultivation technology and enabling multiple and diverse introgressions among wild in domesticated wheat (Civan et al., 2013). Part of this confusion can be clarified by looking at the fragility loci which are more diagnostic than the rest of the genome, being the first hallmarks of domestication (Avni et al., 2017; Nave et al., 2019). Recent genetic evidence shows that a haplotype surrounding a major gene controlling spike fragility (further discussed below) that is found in all the modern tetraploid and hexaploid wheats, originates from wild emmer wheat from the south foothills of Mt. Hermon in the south Levant (Nave et al., 2019). The “where” origin is thus fuzzy, spanning possible sites from the south Levant all the way to the North of the fertile crescent in the upper Euphrates (Figure 2). Nonfragile spikes were initially found together with fragile spikes in early PPNB sites and it took 2–3 millennia for nonbrittleness to be fully dominant among cultivated forms of wheat suggesting that domestication was a gradual process as proposed by Kislev (1984) for emmer wheat and by Tanno and Willcox (2006) for einkorn. The loss of fragility gave rise to the first known domesticated wheat, *T. turgidum* ssp. *dicoccon* which had a hulled grain and a nonbrittle rachis. Free-threshing tetraploid wheat appeared soon following the nonfragile types, in the Near east, late Pre-Pottery Neolithic B (PPNB) (Kislev, 1980; Nesbitt, 2001; Schultze-Motel, 2019).

By the middle PPNB, approximately 9,800 Cal-Y BP, domesticated tetraploid wheat was already found east of the fertile crescent (Riehl et al., 2013), and it is assumed that it came into contact with *Ae. tauschii* to form hexaploid wheat (Feldman, 2001; Zohary et al., 2012). Various biochemical and molecular studies indicated that the most likely area of origin of *T. aestivum* is the south-western corner of the Caspian belt (Nakai, 1979; Jaaska, 1981; Dvorak, 1998). A recent whole-genome analysis of a core collection of 278 accessions covering the eco-geographic distribution of *Ae. tauschii,* in arid and semi-arid habitats from central Asia, Transcaucasia to China, confirmed earlier studies suggesting that the wheat D subgenome is mostly derived from the *strangulata* subgroup originating from the south Caspian Sea and further narrowing down the origin to accessions from the Mazandaran province (Zhou et al., 2021). A recent analysis of 242 *Ae. tauschii* accessions showed that a rare and distinct lineage (different from *strangulata*) from Transcaucasia also contributed ∼1% on average to the current wheat D subgenome (Gaurav et al., 2021), in accordance with earlier studies that analyzed allelic variation of high molecular weight (HMW) glutenins (Giles and Brown, 2006). HMW glutenins from the D subgenome have contributed to wheat baking quality (Orth and Bushuk, 1973; Shewry, 2009). Likewise, the Transcaucasian D lineage contains an HMW glutenin subunit contributing to superior baking quality (Delorean et al., 2021). Hence, a likely scenario is that bread wheat was formed in the south Caspian under domestication, that a few additional introgressions with *strangulata* lines took place such as the *Ae. tauschii* lineage from Transcaucasia, and that there is no true wild *T. aestivum*. Note that there is one semi-wild form of hexaploid wheat, ssp*. tibetanum,* growing in Tibet, whose spike is fragile, hulled and breeds freely with bread wheat (Shao and Li, 1983), but it is generally considered a feral form of bread wheat *T. aestivum* ssp*. aestivum*. A recent study showed that Tibetan wheat became feral through mutations in a local landrace that involved a *Btr1/2* homolog on 3D and *Q* on 5A (Guo et al., 2020). Dvorak et al. (2012) suggested, on the basis of the mapping of the *tenacious glume* (*Tg*) locus in several wheat species, that the BBAA subgenomes of hexaploid wheat originated from a free-threshing tetraploid form and therefore it could not be *T. turgidum* ssp*. dicoccon,* the most ancient domesticated hulled grain tetraploid wheat. This leaves us with the question of the identity of the domesticated tetraploid parent of hexaploid wheat. The free-threshing tetraploid macaroni wheat *T. turgidum* ssp*. durum* is also an unlikely donor since there is no evidence that it existed approximately 8,500 YA when *T. aestivum* was formed. The domesticated free-threshing tetraploid wheat that was cultivated approximately 9,000 YA, starting in the Levant and spreading throughout the fertile crescent, when *T. aestivum* was formed is the primitive tetraploid *T. turgidum* ssp. *parvicoccum* (1980, Kislev, 2009; Schultze-Motel, 2019), making it a candidate as donor of the BBAA genomes. This is reinforced by the fact that the extracted tetraploids produced by Kerber (1964), having the BBAA subgenomes from hexaploid wheat and lacking the D subgenome (see Figure 3), were free threshing and had similar small and oval grain shape as ssp. *parvicoccum* (Kislev, 1980, 2009). While ssp. *parvicoccum* was found in several archaeological sites beyond the fertile crescent, in the Balkans and in Transcaucasia, and as late as approximately 1,000 years ago in Georgia (Schultze-Motel, 2019), it is now extinct as it was replaced during the Roman period by the more prolific and large-grain ssp. *durum* (Kislev, 2009). Therefore, the precise identity of the free-threshing domesticated tetraploid that hybridized with *Ae. tauschii* to form *T. aestivum* remains uncertain. Another puzzling fact about hexaploid wheat origin is that there is no archeological record of the hulled, spelt-like type that should have emerged from a *T. turgidum* × *Ae. tauschii* allohexaploid (see below). The earliest archeological findings of hexaploid wheat are from an 8,400-year-old, free-threshing type in Cafer Hoyuk, upper Euphrates (Bilgic et al., 2016) rather than from the location of origin predicted from the genomic data (Zhou et al., 2021) in the south Caspian (Figure 2). The lack of hulled, spelt-like types in the archeological record suggests that the free-threshing mutation occurred very soon after hexaploid wheat formation and was preferred, in most regions, over the original Spelt-like type. The free-threshing hexaploid wheat settled in central Anatolia and spread rapidly to Europe (Bogaard, 2016).

### Domestication: how?

The brittle rachis phenotype in ssp. *dicoccoides* is controlled by two major loci mapped on the short arm of chromosomes 3A and 3B (Levy and Feldman, 1989; Nalam et al., 2006; Watanabe et al., 2006; Millet et al., 2013). Avni et al. (2017) isolated these two genes, homologous to the barley *Brittle rachis Btr1* and *Btr2* genes (Pourkheirandish et al., 2015). They show that, while a *Btr2* homolog exists in wheat, only a mutation in the *Btr1* orthologs in the wild emmer wheat loci (*br-A1* and *br-B1*) leads to nonbrittleness (Figure 3). The mutations are likely a loss of function: *br-A1* has a 2 bp deletion 290 bases from the start codon and *br-B1* contains a 4-kb TE insertion in its coding region. A mutation in a single homoeoallele, (*br-A1* or *br-B1*) led to partial fragility while the combination of both was needed for the full nonbrittle rachis phenotype. A panel of 113 wild emmer, 85 domestic emmer, and 9 durum lines showed that all domesticated forms carried both mutations (Avni et al., 2017). The accumulation of one mutation after another was thus necessary to reach full nonbrittleness which is consistent with the gradual evolution of this phenotype as seen in the archeological record (Kislev, 1984; Tanno and Willcox, 2006). Moreover, additional QTLs with weaker effects were mapped to other loci (Tzarfati et al., 2014) and might have been selected to further strengthen the rachis. Remarkably, the function and mode of action of the barley *Btr1* and *Btr2* genes or of their wheat homologs remain unknown. In barley, mutations in these genes affect cell wall thickness in the spikelet abscission zone, but not cell wall composition (Pourkheirandish et al., 2015). Nave et al. (2019) investigated the geographical birthplace of the recessive mutations in the *Brittle Rachis* genes *br-A1* and *br-B1*; mutations that determine spike nonbrittleness. They analyzed a core collection of more than 400 lines of wild and domesticated wheat, including hexaploid wheat. They show that mutations in *br-A1* and *br-B1* are of monophyletic origin in the domestic germplasm. They also analyzed the precursor of the domesticated haplotype of *br-A1* in wild accessions and found that it is widespread throughout the Levant, from central Israel to central Turkey. In contrast, the precursor of the domesticated haplotype of *br-B1* was found only in the wild accessions, all from the southern Levant. Moreover, one particular accession from the southern foothills of Mt Hermon carried the haplotype around both *br-A1* and *br-B1* that is found in all domesticated polyploid wheat with the AA and BB genomes (Nave et al., 2019). This suggests a central role for the southern Levant in the domestication of wheat, where one or both mutations might have occurred as opposed to the earlier proposal for the upper Euphrates (Ozkan et al., 2005). In this regard, genetic and archeological evidence do not fully match because the most ancient nonfragile spikes were found in the north Levant at Abu Hureyra and Mureybit (Figure 2). These conflicting conclusions could be reconciled if the eco-geographic distribution of wild wheat drifted during the past 10,000 years since domestication started, or if the archeological record is incomplete.

Concerning hexaploid wheat, one might assume that the hybrid between domesticated free-threshing tetraploid wheat and *Ae*. *tauschii* that gave rise to bread wheat would be fragile due to the wild-type (WT) fragility locus in 3D, *Br-D2* (Figure 3). Fragility in *Ae*. *tauschii* is not taking place at the rachis node, as in ssp. *dicoccoides*, but the breakage occurs below the rachis node (Zohary and Hopf, 2000) so that the spikelet falls with the rachis internode beside it and not below it, as in wild emmer. This fragility trait is controlled by a gene mapping to the long arm of chromosome 3D (Li and Gill, 2006) and designated *Br-D2*, while *Br-A1* and *Br-B1* are located on the short arm, and its mode of action is not clear. At any rate, the spike of the amphiploids between domesticated tetraploid wheat and *Ae*. *tauschii* turned out to be hulled but nonfragile (Figure 3) even though it becomes brittle upon slight pressure indicating co-dominance of the *Br1* and *Br2* genes (McFadden and Sears, 1946). Had it been fragile it might never have been domesticated.

Free threshing is another important domestication trait that is beneficial to the farmer but reduces fitness in the wild. The WT grain is tightly enclosed within the glumes which are large and hard and protect it from birds and grazers while the grain of free-threshing wheat can be easily separated from the glumes. Muramatsu (1986) showed that free threshing emerged at the tetraploid level in an already domesticated nonfragile crop, ssp. *dicoccon* (Figure 3), and that all the free-threshing (or naked) forms of tetraploid wheat carry a dominant allele*,* the *Q* factor, located on the long arm of chromosome 5A (Sears, 1954). The *Q* allele has numerous pleiotropic effects, changing the shape and thickness of the glumes, and leading to a more compact spike and to several other phenotypes (Mac Key, 2005; Simons et al., 2006). Cloning of the *Q* factor showed that it encodes an AP2-like transcription factor, which is consistent with its effect on multiple pathways (Simons et al., 2006). The *q* homoeoalleles underwent pseudogenization on 5B and neofunctionalization on 5D (Zhang et al., 2011). The *Q* mutation is not a duplication as previously thought (Muramatsu, 1963) but a gain-of-function mutation that is found in all the domesticated wheat forms, tetraploid or hexaploid (Simons et al., 2006). Although Simons et al. (2006) considered a SNP, leading to the substitution of valine by isoleucine at position 329, as a possible cause for the *Q* mutation, they also noted a conserved SNP in the miRNA 172 binding site. Subsequent work by Debernardi et al. (2017) showed that in fact, the SNP within the miRNA binding site is the causal polymorphism for the functional difference between the *Q* and *q* alleles. Moreover, *Q* is more abundantly transcribed than WT *q*, which is consistent with its dominant nature, as well as with the disruption of the miRNA 172 suppressive effect (Debernardi et al., 2017) and with the positive dosage effects shown by Muramatsu (1963) who found that extra doses of *q* mimic the effect of *Q* in common wheat. Recently, Zhang et al. (2020b) reported new insights into *Q*’s mode of action using transcriptomics and phenotypic analyses. They show that modification of cell wall thickness and composition of glumes, for example, lignin versus cellulose ratio, correlates with the expression of genes involved in secondary cell wall biosynthesis.

When free-threshing domesticated tetraploid wheat hybridized with the D donor to form bread wheat (Dvorak et al., 2012), we might have expected that the resulting allohexaploid would be free threshing because the *Q* factor is dominant. However, all crosses of either hulled or free-threshing tetraploid wheat with all lines of *Ae. tauschii* yielded only hulled forms resembling ssp. *spelta*, indicating that this subspecies is the prototype of hexaploid wheat (McFadden and Sears, 1946; Kerber and Rowland, 1974). Further analyses showed that the free-threshing *Q*-phenotype was suppressed by the *Tg* gene from *Ae. tauschii,* located on chromosome 2D (*Tg-D1*, Figure 3), and that a single mutation from *Tg* to *tg* produced free-threshing forms (Kerber and Rowland, 1974). The identity of the *Tg* homoeoalleles (*Tg-A1*, *Tg-B1*, and *Tg-D1*) remains unknown. Simonetti et al. (1999), Faris et al. (2014), as well as Sharma et al. (2019), performed fine mapping of the *Tg* loci and showed that dominant homoeoalleles on chromosomes 2A and 2B of emmer wheat (*Tg-A1* and *Tg-B1*) contribute together with the *q* WT allele, to the nonfree-threshing phenotype (Figure 3). They also showed that fully free threshing is achieved by mutations in all three loci and that each mutation has an additive effect on threshability with *Q* having the most profound effect. Free-threshing tetraploid wheats such as *T. turgidum* ssp*. durum*, and possibly ssp. *parvicoccum*, were formed through mutations in all three loci, leading to a *tg-A1tg-A1/tg-B1tg-B1/Q-A1Q-A1* genotype. However, when, where, and in what order these mutations occurred remains unknown. The hulled hexaploid wheat formed through hybridization between free-threshing tetraploid wheat and *Ae. tauschii* was thus expected to have the *Q*/*tg-A1tg-A1/tg-B1tg-B1/Tg-D1Tg-D1* genotype (Figure 3). Such genotypes have not been found among extant hulled wheats. Indeed, most hulled hexaploid spelt wheat lines (*T. aestivum* ssp*. spelta*) examined so far did not have this genotype but rather had a recessive *tg-D1* allele and a dominant *Tg-B1* and *q* allele and are thus thought to be derived from hybridization between free threshing hexaploid wheat with tetraploid hulled emmer wheat (Dvorak et al., 2012). The free-threshing bread wheat, ssp. *aestivum,* with the *Q*/*tg-A1tg-A1/tg-B1tg-B1/tg-D1tg-D1* genotype (Figure 3) must have been preferred by the early farmers of the region and the single mutation in *Tg-D1* enabled the quick replacement of the hulled forms. The isolation of the still elusive *Tg* homoeoalleles will facilitate a better understanding of the evolution of free-threshing tetraploid and hexaploid wheat and the origin of spelt wheat.

In addition to the above-mentioned classical domestication traits, several other traits were selected during the process of domestication. These include plant erectness versus the prostrate grassy types, increased number of seeds per spikelet, increased grain size, and reduced seed dormancy (Feldman, 2001). Overall, the number of domestication-related QTLs mapped to the A subgenome was two-fold higher than those found on the B subgenome, that is, 24 QTL effects for domestication and domestication-related traits in the A subgenome, versus only 11 such QTLs in the B subgenome (Tzarfati et al., 2014). This is in accordance with the concept of “genome asymmetry,” implying that the A subgenome is dedicated to the control of morphological traits, housekeeping metabolic reactions, and yield components (Peng et al., 2003; Feldman et al., 2012).

All of the major evolutionary processes required to produce domesticated tetraploid and hexaploid wheat were completed by the end of the Pre-Pottery Neolithic period in the Near East, about 7,500 BP (Kislev, 1984), and wheat spread away from its Near-East cradle. The appearance and fixation of the nonfragility and free-threshing traits happened mostly within a millennium, between 9,500 and 8,500 years BP for tetraploid and hexaploid wheat. The spread of wheat westward was relatively swift. A surprising finding was the report of wheat DNA in 8,000 years old sediments in a submarine archeological site off the isle of Wight, in the southern UK (Smith et al., 2015). The remarkable transformation and rapid evolution of wheat were probably due to several factors. The allopolyploidy in wheat likely contributed to a highly evolvable nature (Dubcovsky and Dvorak, 2007). While some diploid species such as barley, which has a similar domestication history as wheat, can grow abundantly in a broad range of habitats (see Haas et al. (2019) for a comparison of wheat and Barley domestication and evolution), the wheat diploids grow in limited habitats where they are not the dominant species. In fact, diploid einkorn, which was the first domesticated wheat, did not have the same success as tetraploid wheat, which itself is cultivated approximately 10 times less than hexaploid bread wheat. Intergenomic interactions, described above, together with the impressive capacity of wheat to sustain high rates of mutations (Dvorak and Akhunov, 2005; Akhunov et al., 2007) might have enabled rapid evolution in the allopolyploid background. Moreover, the spread of domesticated tetraploid wheat to new regions facilitated contact between the domesticated and related species or genera, with which they could hybridize and exchange genes. One such encounter is of course the hybridization between domesticated tetraploid wheat and *Ae. Tauschii,* which led to hexaploid wheat formation. While domesticated tetraploid wheats, in keeping with their Near Eastern origin, are adapted to the Mediterranean-type environments (with mild winters and warm, rainless summers), addition of the D subgenome from the central Asiatic *Ae. Tauschii* greatly extended the range of adaptation of hexaploid wheats to a more continental climate over the continental plateaus of Asia and the colder temperate areas in eastern, central, and northern Europe (Feldman, 2001; Zohary et al., 2012). The D genome also contributed resistance to several fungal diseases (Appels and Lagudah, 1990; Yildirim et al., 1995) as well as many desirable grain qualities (Orth and Bushuk, 1973; Delorean et al., 2021) that promoted bread wheat spread and global adoption as the preferred wheat type and the preferred cereal compared to barley.

Major milestones in wheat breeding during the past century, including the green revolution, were recently reviewed (Haas et al., 2019; Venske et al., 2019). The phylogeny of a broad collection of landraces and modern bread wheat lines shows clades that reflect the early east versus west spread of wheat and the adaptation to different climates and in more recent breeding the separation of two major gene pools, one from the western block and one from the Warsaw pact (Pont et al., 2019). Selection for mutations in vernalization (*VRN* genes) that enabled growth under harsh winter conditions or photoperiod sensitivity (e.g. *PPD-D1*) that enabled growth under a broad range of latitude, enabled the expansion of wheat to new areas. The selection of reduced height (*Rht* genes) enabled the green revolution through reduction of lodging under chemical fertilization and high yield.

## The future of wheat evolution

Breeders will continue to rely on wheat biodiversity and wild wheat will keep evolving in nature as they did for the past million years, surviving multiple climatic changes. However, it is not certain that food security can be ensured in the face of anthropogenic activities, nor that biodiversity won’t be affected. Indeed, climate changes, population growth, urbanization, arable land expansion, overgrazing, soil deterioration, etc., might lead to the extinction of several wild wheat populations, to a reduction in biodiversity, and to the emergence of new diseases. Biodiversity conservation in seeds banks and in situ protection are thus essential. Conservation of landraces is mostly taking place in seeds banks, with notable efforts to characterize them genetically (Kilian et al., 2011, 2021; Cavanagh et al., 2013; Dempewolf et al., 2017; Frankin et al., 2020) and to maintain their culture, in situ in farmer’s fields, for niche markets (Negri, 2003). In situ conservation of wild wheat is much more limited. An analysis of a long-term study of in situ conservation of wild emmer wheat showed the resilience, for now, of the genetic structure of a population during the past 36 years in a protected area where temperature raised by ∼1.5°C and CO_2_ concentration by ∼70 ppm (Dahan-Meir et al., 2022). Establishing natural reserves for in situ wild wheat species conservation is critical to ensure their survival and evolution in the wild and to protect biodiversity. Wild emmer wheat (Huang et al., 2016; Klymiuk et al., 2018; Faris et al., 2020; Li et al., 2020) and *Ae. Tauschii* (Bhatta et al., 2018; Gaurav et al., 2021; Zhou et al., 2021) whose chromosomes readily pair with their bread wheat homologs are an invaluable resource for breeders in particular when it comes to biotic and abiotic resistance genes not found in the domestic wheat gene pool. Also, the A subgenome of wild *Triticum* *Timopheevii* and the A genome of *T.* *rartu* are homologous to the A subgenome of bread wheat and can be used as a source of useful genes (Badaeva et al., 2021; Zeibig et al., 2021). The gene pool for wheat breeding can be extended to more distant relatives whose homoeologous chromosomes can recombine with wheat subgenomes in the absence of *Ph1*. In this respect, sequencing the genome of several wheat relatives would provide an invaluable source to facilitate broad gene transfers. The ability to increase the rate of homoeologous recombination in a *ph1* mutant background has been widely used during wheat breeding for the transfer of genes from wild relatives of wheat to the wheat background (Feldman and Sears, 1981). This might be further enhanced by a premeiotic treatment with magnesium in the *ZIP4* (*Ph1* candidate) mutant (Rey et al., 2018) or in the WT background by a virus-induced gene silencing treatment of *Ph1* candidates (Bhullar et al., 2014). During the 20^th^ century, wheat geneticists and breeders have transferred genes from at least 52 species whose chromosomes do not pair regularly with domesticated wheat chromosomes (*Aegilops*, *Agropyron*, *Ambylopyrum*, *Dasypyrum*, *Elymus*, *Hordeum*, *Leymus*, *Lophopyrum*, *Psathyrostachys*, *Pseudoroegneria*, *Secale*, *Thinopyrum*, and *Triticum*; Wulff and Moscou, 2014). Several contemporary introgressions from wild relatives were precisely mapped following de novo sequencing of multiple wheat lines (Walkowiak et al., 2020; Keilwagen et al., 2022). The main motivation for these wide transfers was for both resistance to diseases and tolerance to various abiotic stresses. Improving our ability to perform wide gene transfer precisely and effectively is thus critical. The new technologies of genome editing, shown to work well in wheat (Gao, 2021; Li et al., 2021), offer the promise of inducing new targeted genetic variation in wheat in the near future. The mapping of important QTLs in a broad germplasm, rich datasets of genomes and transcriptome sequencing, future data on proteomics and metabolomics, computational tools including Artificial Intelligence, together with speed-breeding (Watson et al., 2018), will enable breeding wheat more efficiently, including locally adapted varieties, to face the challenges of climate change (Xiong et al., 2021). In summary, we can expect that breeding will accelerate to create new varieties that will contain new genetic variants, either induced or from wild wheat relatives.

## Notes

Both authors contributed to writing this article.

The authors responsible for distribution of materials integral to the findings presented in this article in accordance with the policy described in the Instructions for Authors (https://academic.oup.com/plcell) are: Avraham A. Levy(avi.levy@weizmann.ac.il) and Moshe Feldman (moshe.feldman@weizmann.ac.il).

## References

[koac130-B2] Aaronsohn A (1910) Agricultural and botanical explorations in Palestine. Bull Plant Ind 180: 1–63

[koac130-B3] Akhunov ED , Akhunova AR, Dvorak J (2007) Mechanisms and rates of birth and death of dispersed duplicated genes during the evolution of a multigene family in diploid and tetraploid wheats. Mol Biol Evol 24: 539–550 17135334 10.1093/molbev/msl183

[koac130-B4] Akhunov ED , Akhunova AR, Linkiewicz AM, Dubcovsky J, Hummel D, Lazo G, Chao SM, Anderson OD, David J, Qi LL, et al (2003) Synteny perturbations between wheat homoeologous chromosomes caused by locus duplications and deletions correlate with recombination rates. Proc Natl Acad Sci USA 100: 10836–10841 12960374 10.1073/pnas.1934431100 PMC196889

[koac130-B5] Appels R , Honeycutt RL (1986) rDNA: evolution over a billion years. *In* Dutta SK, ed, DNA Systematics. CRC Press, Boca Raton, FL, pp 81–135

[koac130-B6] Appels R , Lagudah ES (1990) Manipulation of chromosomal segments from wild wheat for the improvement of bread wheat. Austral J Plant Physiol 17: 253–266

[koac130-B7] Attia T , Ekingen H, Robbelen G (1979) origin of 3D-suppressor of homoeologous pairing in hexaploid wheat. Z. Pflanzenzücht 83: 131–126

[koac130-B8] Avni R , Lux T, Minz-Dub A, Millet E, Sela H, Distelfeld A, Deek J, Yu G, Steuernagel B, Pozniak C, et al (2021) Genome sequences of *Aegilops* species of section Sitopsis reveal phylogenetic relationships and provide resources for wheat improvement. Plant J 110: 179–192 10.1111/tpj.15664 PMC10138734 34997796

[koac130-B9] Avni R , Nave M, Barad O, Baruch K, Twardziok SO, Gundlach H, Hale I, Mascher M, Spannagl M, Wiebe K, et al (2017) Wild emmer genome architecture and diversity elucidate wheat evolution and domestication. Science 357: 93–97 28684525 10.1126/science.aan0032

[koac130-B10] Badaeva E , Konovalov F, Knuepffer H, Fricano A, Ruban A, Kehel Z, Zoshchuk S, Surzhikov S, Neumann K, Graner A, et al (2021) Genetic diversity, distribution and domestication history of the neglected GGAtAt genepool of wheat. Theor Appl Genet 134: 3493 34379147 10.1007/s00122-021-03931-x PMC8440298

[koac130-B11] Bariah I , Keidar-Friedman D, Kashkush K (2020) Where the wild things are: transposable elements as drivers of structural and functional variations in the wheat genome. Front Plant Sci 11: 585515 33072155 10.3389/fpls.2020.585515 PMC7530836

[koac130-B12] Bar-Yosef O (1998) On the nature of transitions: the middle to upper Palaeolithic and the Neolithic revolution. Cambridge Archaeol J 8: 141–163

[koac130-B13] Baum BR , Feldman M (2010) Elimination of 5S DNA unit classes in newly formed allopolyploids of the genera *Aegilops* and *Triticum.* Genome 53: 430–438 20555432 10.1139/g10-017

[koac130-B14] Bennetzen JL (2005) Transposable elements, gene creation and genome rearrangement in flowering plants. Curr Opin Genet Dev 15: 621–627 16219458 10.1016/j.gde.2005.09.010

[koac130-B15] Bernhardt N , Brassac J, Dong X, Willing E, Poskar C, Kilian B, Blattner F (2020) Genome‐wide sequence information reveals recurrent hybridization among diploid wheat wild relatives. Plant J 102: 493–506 31821649 10.1111/tpj.14641

[koac130-B16] Bhatta M , Morgounov A, Belamkar V, Baenziger PL, Poland J (2018) Unlocking the novel genetic diversity and population structure of synthetic Hexaploid wheat. BMC Genomics 19: 591 30081829 10.1186/s12864-018-4969-2 PMC6090860

[koac130-B17] Bhullar R , Nagarajan R, Bennypaul H, Sidhu GK, Sidhu G, Rustgi S, Von Wettstein D, Gill KS (2014) Silencing of a metaphase I-specific gene results in a phenotype similar to that of the pairing homeologous 1 (*Ph1*) gene mutations. Proc Natl Acad Sci USA 111: 14187–14192 25232038 10.1073/pnas.1416241111 PMC4191769

[koac130-B18] Bilgic H , Hakki EE, Pandey A, Khan MK, Akkaya MS (2016) Ancient DNA from 8400 year-old catalhoyuk wheat: implications for the origin of neolithic agriculture. PLoS One 11: e0151974 26998604 10.1371/journal.pone.0151974 PMC4801371

[koac130-B19] Birchler JA , Veitia RA (2021) One hundred years of gene balance: how stoichiometric issues affect gene expression, genome evolution, and quantitative traits. Cytogenet Genome Res 161: 1–22 10.1159/000519592 34814143

[koac130-B20] Bogaard A (2016) Archaeobotany: the wheat and the chaff. Nat Plants 2: 16079 27255842 10.1038/nplants.2016.79

[koac130-B21] Bolot S , Abrouk M, Masood-Quraishi1 U, Stein N, Messing J, Feuillet C, Salse J (2009) The ‘inner circle’ of the cereal genomes. Curr Opin Plant Biol 12: 119–125 19095493 10.1016/j.pbi.2008.10.011

[koac130-B22] Bomblies K , Weigel D (2007) Hybrid necrosis: autoimmunity as a potential gene-flow barrier in plant species. Nat Rev Genet 8: 382–393 17404584 10.1038/nrg2082

[koac130-B23] Caldwell KS , Dvorak J, Lagudah ES, Akhunov E, Luo MC, Wolters P, Powell W (2004) Sequence polymorphism in polyploid wheat and their D-genome diploid ancestor. Genetics 167: 941–947 15238542 10.1534/genetics.103.016303 PMC1470897

[koac130-B24] Cavanagh CR , Chao S, Wang S, Huang BE, Stephen S, Kiani S, Forrest K, Saintenac C, Brown-Guedira GL, Akhunova A, et al (2013) Genome-wide comparative diversity uncovers multiple targets of selection for improvement in hexaploid wheat landraces and cultivars. Proc Natl Acad Sci USA 110: 8057–8062 23630259 10.1073/pnas.1217133110 PMC3657823

[koac130-B25] Cheng ZJ , Murata M (2003) A centromeric tandem repeat family originating from a part of *Ty3*/gypsy retroelement in wheat and its relatives. Genetics 164: 665–672 12807787 10.1093/genetics/164.2.665 PMC1462596

[koac130-B26] Choulet F , Wicker T, Rustenholz C, Paux E, Salse J, Leroy P, Schlub S, Le Paslier MC, Magdelenat G, Gonthier C, et al (2010) Megabase level sequencing reveals contrasted organization and evolution patterns of the wheat gene and transposable element spaces. Plant Cell 22: 1686–1701 20581307 10.1105/tpc.110.074187 PMC2910976

[koac130-B27] Civan P , Ivanicova Z, Brown TA (2013) Reticulated origin of domesticated emmer wheat supports a dynamic model for the emergence of agriculture in the Fertile Crescent. PLoS One 8: e81955 24312385 10.1371/journal.pone.0081955 PMC3843696

[koac130-B28] Conant GC , Birchler JA, Pires JC (2014) Dosage, duplication, and diploidization: clarifying the interplay of multiple models for duplicate gene evolution over time. Curr Opin Plant Biol 19: 91–98 24907529 10.1016/j.pbi.2014.05.008

[koac130-B29] Dahan Meir T , Ellis TJ, Mafessoni F, Sela H, Manisterski J, Avivi-Ragolsky N, Raz A, Feldman M, Anikster Y, Nordborg M, et al (2022) The genetic structure of a wild wheat population has remained associated with microhabitats over 36 years bioRxiv 2022.01.10.475641; 10.1101/2022.01.10.475641

[koac130-B30] Darrier B , Rimbert H, Balfourier F, Pingault L, Josselin AA, Servin B, Navarro J, Choulet F, Paux E, Sourdille P (2017) High-resolution mapping of crossover events in the hexaploid wheat genome suggests a universal recombination mechanism. Genetics 206: 1373–1388 28533438 10.1534/genetics.116.196014 PMC5500137

[koac130-B31] Darwin CR (1868) The Variation of Animals and Plants Under Domestication. John Murray, London

[koac130-B32] Debernardi JM , Lin H, Chuck G, Faris JD, Dubcovsky J (2017) microRNA172 plays a crucial role in wheat spike morphogenesis and grain threshability. Development 144: 1966–1975 28455375 10.1242/dev.146399 PMC5482987

[koac130-B33] Delorean E , Gao L, Lopez JFC, Open Wild Wheat Consortium, Wulff BBH, Ibba MI, Poland J (2021) High molecular weight glutenin gene diversity in Aegilops tauschii demonstrates unique origin of superior wheat quality. Commun Biol **4**: 1242 10.1038/s42003-021-02563-7 PMC8560932 34725451

[koac130-B34] Dempewolf H , Baute G, Anderson J, Kilian B, Smith C, Guarino L (2017) Past and Future Use of Wild Relatives in Crop Breeding. Crop Sci 57: 1070–1082

[koac130-B35] Dubcovsky J , Dvorak J (2007) Genome plasticity a key factor in the success of polyploid wheat under domestication. Science 316: 1862–1866 10.1126/science.1143986 17600208 10.1126/science.1143986 PMC4737438

[koac130-B36] Dvorak J (1998) Genome analysis in the *Triticum*-*Aegilops* alliance. *In* Slinkard AE, ed, Proceedings of the 9th International Wheat Genetics Symposium. University Extension Press, University of Saskatoon, Saskatoon, Saskatchewan, Canada, pp 8–11

[koac130-B37] Dvorak J (2009) Triticeae genome structure and evolution. *In* Feuiller C, Muehlbauer GJ, eds, Genetics and Genomics of the Triticeae, Plant Genetics and Genomics: Crops and Models, Vol 7. Springer, Berlin, Germany, pp 685–711

[koac130-B38] Dvorak J , Akhunov ED (2005) Tempos of gene locus deletions and duplications and their relationship to recombination rate during diploid and polyploid evolution in the Aegilops-Triticum alliance. Genetics 171: 323–332 15996988 10.1534/genetics.105.041632 PMC1456522

[koac130-B39] Dvorak J , Akhunov ED, Akhunov AR, Deal KR, Luo MC (2006) Molecular characterization of a diagnostic DNA marker for domesticated tetraploid wheat provides evidence for gene flow from wild tetraploid wheat to hexaploid wheat. Mol Biol Evol 23: 1386–1396 16675504 10.1093/molbev/msl004

[koac130-B40] Dvorak J , Deal KR, Luo M-C, You FM, von Borstel K, Dehghani H (2012) The origin of spelt and free-threshing hexaploid wheat. J Hered 103: 426–441 22378960 10.1093/jhered/esr152

[koac130-B41] Eilam T , Anikster Y, Millet E, Manisterski J, Feldman M (2008) Nuclear DNA amount and genome downsizing in natural and synthetic allopolyploids of the genera *Aegilops* and *Triticum*. Genome 51: 616–627 18650951 10.1139/G08-043

[koac130-B42] Erayman M , Sandhu D, Sidhu D, Dilbirligi M, Baenziger PS, Gill KS (2004) Demarcating the gene-rich regions of the wheat genome. Nucleic Acids Res 32: 3546–3565 15240829 10.1093/nar/gkh639 PMC484162

[koac130-B43] FAO (2021) FAO cereal supply and demand brief. http://www.fao.org/worldfoodsituation/csdb/en/

[koac130-B44] Faris JD , Haen KM, Gill BS (2000) Saturation mapping of a gene-rich recombination hot spot region in wheat. Genetics 154: 823–835 10655233 10.1093/genetics/154.2.823 PMC1460934

[koac130-B45] Faris JD , Overlander ME, Kariyawasam GK, Carter A, Xu SS, Liu Z (2020) Identification of a major dominant gene for race-nonspecific tan spot resistance in wild emmer wheat. Theor Appl Genet 133: 829–841 31863156 10.1007/s00122-019-03509-8

[koac130-B46] Faris JD , Zhang Z, Chao S (2014) Map-based analysis of the tenacious glume gene *Tg-B1* of wild emmer and its role in wheat domestication. Gene 542: 198–208 24657062 10.1016/j.gene.2014.03.034

[koac130-B47] Fedoroff NV (2012) Presidential address. Transposable elements, epigenetics, and genome evolution. Science 338: 758–767 23145453 10.1126/science.338.6108.758

[koac130-B48] Feldman M (1988) Cytogenetic and molecular approaches to alien gene transfer in wheat. *In* TE Miller, RMD Koebner, eds, Proceedings of the 7th International Wheat Genetics Symposium, Vol. 1. Cambridge, England, pp 23–32

[koac130-B49] Feldman M (2001) The origin of cultivated wheat. *In* Bonjean A, Angus W, eds, The World Wheat Book. Lavoisier Tech. & Doc., Paris

[koac130-B50] Feldman M , Levy AA (2012) Genome evolution due to allopolyploidization in wheat. Genetics 192: 763–774 23135324 10.1534/genetics.112.146316 PMC3522158

[koac130-B51] Feldman M , Levy AA, Fahima T, Korol A (2012) Genome asymmetry in allopolyploid plants - wheat as a model. J Exp Bot 63: 5045–5059 22859676 10.1093/jxb/ers192

[koac130-B52] Feldman M , Liu B, Segal G, Abbo S, Levy AA, Vega JM (1997) Rapid elimination of low-copy DNA sequences in polyploid wheat: a possible mechanism for differentiation of homoeologous chromosomes. Genetics 147: 1381–1387 9383078 10.1093/genetics/147.3.1381 PMC1208259

[koac130-B53] Feldman M , Sears ER (1981) The wild gene resources of wheat. Sci Am 244: 102–112

[koac130-B54] Feuillet C , Keller B (1999) High gene density is conserved at syntenic loci of small and large grass genomes. Proc Natl Acad Sci USA 96: 8265–8270 10393983 10.1073/pnas.96.14.8265 PMC22223

[koac130-B55] Flavell RB , O’Dell M (1979) The genetic control of nucleolus formation in wheat. Chromosoma 71: 135–152

[koac130-B56] Frankin S , Kunta S, Abbo S, Sela H, Goldberg BZ, Bonfil DJ, Levy AA, Avivi-Ragolsky N, Nashef K, Roychowdhury R, et al (2020) The Israeli-Palestinian wheat landraces collection: restoration and characterization of lost genetic diversity. J Sci Food Agric 100: 4083–4092 31141162 10.1002/jsfa.9822

[koac130-B57] Fukui KN , Suzuki G, Lagudah ES, Rahman S, Appels R, Yamamoto M, Mukai Y (2001) Physical arrangement of retrotransposon-related repeats in centromeric regions of wheat. Plant Cell Physiol 42: 189–119 11230573 10.1093/pcp/pce026

[koac130-B58] Galili G , Levy AA, Feldman M (1986) Gene-dosage compensation of endosperm proteins in hexaploid wheat *Triticum aestivum*. Proc Natl Acad Sci USA 83: 6524–6528 16593753 10.1073/pnas.83.17.6524 PMC386536

[koac130-B59] Gao C (2021) Genome engineering for crop improvement and future agriculture. Cell 184: 1621–1635 33581057 10.1016/j.cell.2021.01.005

[koac130-B60] Gardiner LJ , Wingen LU, Bailey P, Sánchez-Martín J, Horsnell R, Gao L, Brar GS, Widrig V, Raupp WJ, Singh N, et al (2019) Analysis of the recombination landscape of hexaploid bread wheat reveals genes controlling recombination and gene conversion frequency. Genome Biol 20: 69 30982471 10.1186/s13059-019-1675-6 PMC6463664

[koac130-B61] Gaurav K , Arora S, Silva P, Sanchez-Martin J, Horsnell R, Gao L, Brar GS, Widrig V, John Raupp W, Singh N, et al (2021) Population genomic analysis of *Aegilops tauschii* identifies targets for bread wheat improvement. Nat Biotechnol 10: 422–431 10.1038/s41587-021-01058-4 PMC8926922 34725503

[koac130-B63] Giles RJ , Brown TA (2006) GluDy allele variations in Aegilops tauschii and Triticum aestivum: implications for the origins of hexaploid wheats. Theor Appl Genet 112: 1563–1572 16568284 10.1007/s00122-006-0259-5

[koac130-B64] Gill KS , Gill BS (1991) A DNA fragment mapped within the submicroscopic deletion of *ph1*, a chromosome pairing regulator gene in polyploid wheat. Genetics 129: 257–259 1936962 10.1093/genetics/129.1.257 PMC1204574

[koac130-B65] Glémin S , Scornavacca C, Dainat J, Burgarella C, Viader V, Ardisson M, Sarah G, Santoni S, David J, Ranwez V (2019) Pervasive hybridizations in the history of wheat relatives. Sci Adv 5: eaav9188 31049399 10.1126/sciadv.aav9188 PMC6494498

[koac130-B66] Griffiths S , Sharp R, Foote TN, Bertin I, Wanous M, Reader S, Colas I, Moore G (2006) Molecular characterization of *Ph1* as a major chromosome pairing locus in polyploid wheat. Nature 439: 749–752 16467840 10.1038/nature04434

[koac130-B67] Guo X , Han F (2014) Asymmetric epigenetic modification and elimination of rDNA sequences by polyploidization in wheat^.^ Plant Cell 26: 4311–4327 25415973 10.1105/tpc.114.129841 PMC4277213

[koac130-B68] Guo W , Xin M, Wang Z, Yao Y, Hu Z, Song W, Yu K, Chen Y, Wang X, Guan P, et al (2020) Origin and adaptation to high altitude of Tibetan semi-wild wheat. Nat Commun 11: 5085 33033250 10.1038/s41467-020-18738-5 PMC7545183

[koac130-B69] Haas M , Schreiber M, Mascher M (2019) Domestication and crop evolution of wheat and barley: genes, genomics, and future directions. J Integr Plant Biol 61: 204–225 30414305 10.1111/jipb.12737

[koac130-B70] Han FP , Fedak G, Guo WL, Liu B (2005) Rapid and repeatable elimination of a parental genome specific DNA repeat (pGcIR-1a) in newly synthesized wheat allopolyploids. Genetics 170: 1239–1245 15911583 10.1534/genetics.104.039263 PMC1451191

[koac130-B71] Han FP , Fedak G, Ouellet T, Liu B (2003) Rapid genomic changes in interspecific and intergeneric hybrids and allopolyploids of Triticeae. Genome 46: 716–723 12897878 10.1139/g03-049

[koac130-B72] Handa H , Kanamori H, Tanaka T, Murata K, Kobayashi F, Robinson SJ, Koh CS, Pozniak CJ, Sharpe AG, Paux E, et al (2018) Structural features of two major nucleolar organizer regions (NORs), Nor-B1 and Nor-B2, and chromosome-specific rRNA gene expression in wheat. Plant J 96: 1148–1159 30238531 10.1111/tpj.14094

[koac130-B73] He F , Pasam R, Shi F, Kant S, Keeble-Gagnere G, Kay P, Forrest K, Fritz A, Hucl P, Wiebe K, et al (2019) Exome sequencing highlights the role of wild-relative introgression in shaping the adaptive landscape of the wheat genome. Nat Genet 51: 896–904 31043759 10.1038/s41588-019-0382-2

[koac130-B74] Huang L , Raats D, Sela H, Klymiuk V, Lidzbarsky G, Feng L, Krugman T, Fahima T (2016) Evolution and adaptation of wild emmer wheat populations to biotic and abiotic stresses. Annu Rev Phytopathol 54: 279–301 27296141 10.1146/annurev-phyto-080614-120254

[koac130-B75] International Wheat Genome Sequencing Consortium (IWGSC) (2018) Shifting the limits in wheat research and breeding using a fully annotated reference genome. Science 361: eaar7191 30115783 10.1126/science.aar7191

[koac130-B76] Jaaska V (1981) Aspartate aminotransferase and alcohol dehydrogenase isoenzyme: intraspecific differentiation in *Aegilops tauschii* the origin of the D genome polyploids in the wheat group. Plant Syst Evol 137: 259–273

[koac130-B77] Jia J , Zhao S, Kong X, Li Y, Zhao G, He W, Appels R, Pfeifer M, Tao Y, Zhang X, et al (2013) Aegilops tauschii draft genome sequence reveals a gene repertoire for wheat adaptation. Nature 496: 91–95 23535592 10.1038/nature12028

[koac130-B78] Jiang N , Bao Z, Zhang X, Eddy SR, Wessler SR (2004) Pack-MULE transposable elements mediate gene evolution in plants. Nature 431: 569–573 15457261 10.1038/nature02953

[koac130-B79] Jiang J , Gill BS (1994) New 18S-26S ribosomal RNA gene loci: chromosomal landmarks for the evolution of polyploid wheats. Chromosoma 103: 179–185 7924620 10.1007/BF00368010

[koac130-B80] Kashkush K , Feldman M, Levy AA (2002) Gene loss, silencing and activation in a newly synthesized wheat allotetraploid. Genetics 160: 1651–1659 11973318 10.1093/genetics/160.4.1651 PMC1462064

[koac130-B81] Kashkush K , Feldman M, Levy AA (2003) Transcriptional activation of retrotransposons alters the expression of adjacent genes in wheat. Nat Genet 33: 102–106 12483211 10.1038/ng1063

[koac130-B82] Keidar D , Doron C, Kashkush K (2018) Genome-wide analysis of a recently active retrotransposon, Au SINE, in wheat: content, distribution within subgenomes and chromosomes, and gene associations. Plant Cell Rep 37: 193–208 29164313 10.1007/s00299-017-2213-1 PMC5787218

[koac130-B83] Keidar-Friedman D , Bariah I, Kashkush K (2018) Genome-wide analyses of miniature inverted-repeat transposable elements reveals new insights into the evolution of the Triticum-Aegilops group. PLoS One 13: e0204972 30356268 10.1371/journal.pone.0204972 PMC6200218

[koac130-B84] Keilwagen J , Lehnert H, Berner T, Badaeva E, Himmelbach A, Börner A, Kilian B (2022) Detecting major introgressions in wheat and their putative origins using coverage analysis. Sci Rep **12**: 1908. 10.21203/rs.3.rs-910879/v1 PMC8813953 35115645

[koac130-B85] Kenan-Eichler M , Leshkowitz D, Tal L, Noor E, Melamed-Bessudo C, Feldman M, Levy AA (2011) Wheat hybridization and polyploidization results in deregulation of small RNAs. Genetics 188: 263–272 21467573 10.1534/genetics.111.128348 PMC3122319

[koac130-B86] Kerber ER (1964) Wheat: reconstitution of the tetraploid component (AABB) of hexaploids. Science 143: 253–255 17753152 10.1126/science.143.3603.253

[koac130-B87] Kerber ER , Rowland GG (1974) Origin of the free threshing character in hexaploid wheat. Can J Genet Cytol 16: 145–154

[koac130-B88] Kidwell MG , Lisch D (1997) Transposable elements as sources of variation in animals and plants. Proc Natl Acad Sci USA 94: 7704–7711 9223252 10.1073/pnas.94.15.7704 PMC33680

[koac130-B89] Kihara H (1919) Uber cytologische Studien bei einigen Getreidiarten. I. Species-bastarde des weizens und weizenroggen-bastarde. Bot Mag Tokyo 32: 17–38

[koac130-B90] Kihara H (1924) Cytologische und Genetische Studien bei Wichtigcn Getreidearten mit Besonderer Rücksicht ouf das Verhalten der Chromosomen und die Sterilitat in den Bastarden. Memoirs of the College of Science, Kyoto imperial University, Kyoto, Japan, pp 1–200

[koac130-B91] Kihara H (1944) Discovery of the DD-analyser, one of the ancestors of *Triticum vulgare*. Agric Hortic 19: 13–14

[koac130-B92] Kilian B , Dempewolf H, Guarino L, Werner P, Coyne C, Warburton ML (2021) Crop Science special issue: adapting agriculture to climate change: a walk on the wild side. Crop Sci 61: 32–36.

[koac130-B93] Kilian B , Knupffer H, Hammer K (2014) Elisabeth Schiemann (1881–1972): a pioneer of crop plant research, with special reference to cereal phylogeny. Genet Resour Crop Ev 61: 89–106

[koac130-B94] Kilian B , Mammen K, Millet E, Sharma R, Graner A, Salamini F, Hammer K, Özkan H (2011) Aegilops L. *In* Kole C, ed, Wild Crop Relatives: Genomic and Breeding Resources Cereals. Springer, Berlin, Germany, pp 1–76.

[koac130-B95] Kislev ME (1980) *Triticum parvicoccum* sp. nov, the oldest naked wheat. Isr J Bot 28: 95–107

[koac130-B96] Kislev ME (1984) Emergence of wheat agriculture. Paleorient 10: 61–70

[koac130-B97] Kislev ME (2009) Reconstructing the ear morphology of ancient small-grain wheat (*Triticum turgidum* ssp. *parvicoccum)**. In* Fairbairn A, Weiss E, eds, From Foragers to Farmers: Papers in Honour of Gordon C Hillman. Oxbow Books, England, pp 235–238

[koac130-B98] Klymiuk V , Yaniv E, Huang L, Raats D, Fatiukha A, Chen S, Feng L, Frenkel Z, Krugman T, Lidzbarsky G, et al (2018) Cloning of the wheat Yr15 resistance gene sheds light on the plant tandem kinase-pseudokinase family. Nat Commun 9: 3735 30282993 10.1038/s41467-018-06138-9 PMC6170490

[koac130-B99] Levy AA , Feldman M (1989) Genetics of morphological traits in wild wheat, *Triticum turgidum var. dicoccoides*. Euphytica 40: 275–281

[koac130-B100] Li M , Dong L, Li B, Wang Z, Xie J, Qiu D, Li Y, Shi W, Yang L, Wu Q, et al (2020) A CNL protein in wild emmer wheat confers powdery mildew resistance. New Phytol 228: 1027–1037 32583535 10.1111/nph.16761

[koac130-B101] Li W , Gill B (2006) Multiple genetic pathways for seed shattering in the grasses. Funct Integr Genomics 6: 300–309 16404644 10.1007/s10142-005-0015-y

[koac130-B102] Li T , Hu J, Sun Y, Li B, Zhang D, Li W, Liu J, Li D, Gao C, Zhang Y, et al (2021) Highly efficient heritable genome editing in wheat using an RNA virus and bypassing tissue culture. Mol Plant 14: 1787–1798 34274523 10.1016/j.molp.2021.07.010

[koac130-B103] Li LF , Zhang ZB, Wang ZH, Li N, Sha Y, Wang XF, Ding N, Li Y, Zhao J, Wu Y, et al (2022) Genome sequences of the five *Sitopsis* species of *Aegilops* and the origin of polyploid wheat B subgenome. Mol Plant 15: 488–503 34979290 10.1016/j.molp.2021.12.019

[koac130-B104] Liu B , Vega JM, Feldman M (1998) Rapid genomic changes in newly synthesized amphiploids of *Triticum* and *Aegilops*. II. Changes in low-copy coding DNA sequences. Genome 41: 535–542 9796102 10.1139/g98-052

[koac130-B105] Liu Y , Yuan J, Jia G, Ye W, Chen ZJ, Song Q (2021) Histone H3K27 dimethylation landscapes contribute to genome stability and genetic recombination during wheat polyploidization. Plant J 105: 678–690 33131144 10.1111/tpj.15063

[koac130-B106] Luo MC , Deal KR, Akhunov ED, Akhunova AR, Anderson OD, Anderson JA, Blake N, Clegg MT, Coleman-Derr D, Conley EJ, et al (2009) Genome comparisons reveal a dominant mechanism of chromosome number reduction in grasses and accelerated genome evolution in Triticeae. Proc Natl Acad Sci USA 106: 15780–15785 19717446 10.1073/pnas.0908195106 PMC2747195

[koac130-B107] Luo MC , Gu YQ, Puiu D, Wang H, Twardziok SO, Deal KR, Huo N, Zhu T, Wang L, Wang Y, et al (2017) Genome sequence of the progenitor of the wheat D genome *Aegilops tauschii*. Nature 551: 498–502 29143815 10.1038/nature24486 PMC7416625

[koac130-B108] Luo MC , Gu YQ, You FM, Deal KR, Ma Y, Hu Y, Huo N, Wang Y, Wang J, Chen S, et al (2013) A 4-gigabase physical map unlocks the structure and evolution of the complex genome of *Aegilops tauschii*, the wheat D-genome progenitor. Proc Natl Acad Sci USA 110: 7940–7945 23610408 10.1073/pnas.1219082110 PMC3651469

[koac130-B109] Luo MC , Yang ZL, You FM, Kawahara T, Waines JG, Dvorak J (2007) The structure of wild and domesticated emmer wheat populations, gene flow between them, and the site of emmer domestication. Theor Appl Genet 114: 947–959 17318496 10.1007/s00122-006-0474-0

[koac130-B110] Ma XF , Gustafson JP (2006) Timing and rate of genome variation in triticale following allopolyploidization. Genome 49: 950–958 17036070 10.1139/g06-078

[koac130-B111] Mac Key J (1954) The taxonomy of hexaploid wheat. Svensk Bot Tidskr 48: 579–590

[koac130-B112] Mac Key J (2005) Wheat: its concept, evolution and taxonomy. *In* Royo C, Di Fonzo N, eds, Durum Wheat, Current Approaches, Future Strategies, Vol. 1. CRC Press, Boca Raton, FL, pp 3–61

[koac130-B113] Maccaferri M , Harris NS, Twardziok SO, Pasam RK, Gundlach H, Spannagl M, Ormanbekova D, Lux T, Prade VM, Milner SG, et al (2019) Durum wheat genome highlights past domestication signatures and future improvement targets. Nat Genet 51: 885–895 30962619 10.1038/s41588-019-0381-3

[koac130-B114] Marcussen T , Sandve SR, Heier L, Spannagl M, Pfeifer M, Jakobsen KS, Wulff BBH, Steuernagel B, Klaus FX, Mayer KFX, et al (2014) Ancient hybridizations among the ancestral genomes of bread wheat. Science 345: 288–291 25035499 10.1126/science.1250092

[koac130-B115] Mayrose I , Zhan SH, Rothfels CJ, Magnuson-Ford K, Barker MS, Rieseberg LH, Otto SP (2011) Recently formed polyploid plants diversify at lower rates. Science 333: 1257 21852456 10.1126/science.1207205

[koac130-B116] McClintock B (1934) The relation of a particular chromosomal element to the development of the nucleoli in Zea mays. Zeit Zellforsch Mik Anat 21: 294–328

[koac130-B117] McFadden ES , Sears ER (1944) The artificial synthesis of *Triticum spelta*. Records Genet Soc Amer 13: 26–27

[koac130-B118] McFadden ES , Sears ER (1946) The origin of *Triticum spelta* and its free-threshing hexaploid relatives. J Hered 37: 81–89, 107–116 20985728 10.1093/oxfordjournals.jhered.a105590

[koac130-B119] Miller TE , Hutchinson J, Reader SM (1983) The identification of the nucleolus organizer chromosomes of diploid wheat. Theor Appl Genet 65: 145–147 24263342 10.1007/BF00264881

[koac130-B120] Millet E , Rong JK, Qualset CO, McGuire PE, Bernard M, Sourdille P, Feldman M (2013) Production of chromosome-arm substitution lines of wild emmer in common wheat. Euphytica 190: 1–17

[koac130-B121] Molinier J , Ries G, Bonhoeffer S, Hohn B (2004) Interchromatid and Interhomolog Recombination in Arabidopsis thaliana. Plant Cell 16: 342–352 14729918 10.1105/tpc.019042 PMC341908

[koac130-B122] Moore G , Devos KM, Wang Z, Gale MD (1995) Cereal genome evolution. Grasses, line up and form a circle. Curr Biol 5: 737–739 7583118 10.1016/s0960-9822(95)00148-5

[koac130-B123] Morgante M , Brunner S, Pea G, Fengler K, Zuccolo A, Rafalski A (2005) Gene duplication and exon shuffling by helitron-like transposons generate intraspecies diversity in maize. Nat Genet 37: 997–1002 16056225 10.1038/ng1615

[koac130-B124] Muramatsu M (1963) Dosage effect of the spelta gene *q* of hexaploid wheat. Genetics 48: 469–482 17248158 10.1093/genetics/48.4.469 PMC1210486

[koac130-B125] Muramatsu M (1986) The *vulgare* super gene, *Q*: its universality in *durum* wheat and its phenotypic effects in tetraploid and hexaploid wheats. Can J Genet Cytol 28: 30–41

[koac130-B126] Murat F , Armero A, Pont C, Klopp C, Salse J (2017) Reconstructing the genome of the most recent common ancestor of flowering plants. Nat Genet 49: 490–496 28288112 10.1038/ng.3813

[koac130-B127] Murat F , Pont C, Salse J (2014) Paleogenomics in Triticeae for translational research. Curr Plant Biol 1: 34–39

[koac130-B128] Nakai Y (1979) Isozyme variation in *Aegilops* and *Triticum*. IV. The origin of the common wheats revealed from the study on esterase isozymes in synthesized wheats. Jpn J Genet 54: 175–189

[koac130-B129] Nalam VJ , Vales MI, Watson CJW, Kianian SF, Riera-Lizarazu O (2006) Map-based analysis of genes affecting the brittle rachis character in tetraploid wheat (*Triticum turgidum* L.). Theor Appl Gen 112: 373–381 10.1007/s00122-005-0140-y 16328232

[koac130-B130] Nave M , Avni R, Çakır E, Portnoy V, Sela H, Pourkheirandish M, Ozkan H, Hale I, Takao Komatsuda T, et al (2019) Wheat domestication in light of haplotype analyses of the Brittle rachis 1 genes (*BTR1-A* and *BTR1-B*). Plant Sci 285 193–199 31203884 10.1016/j.plantsci.2019.05.012

[koac130-B131] Negri V (2003) Landraces in central Italy: where and why they are conserved and perspectives for their on-farm conservation. Genet Resourc Crop Evol 50: 871–885

[koac130-B132] Nesbitt M (2001) Wheat evolution: integrating archaeological and biological evidence. In Caligari P.D.S., Brandham PE eds, Wheat Taxonomy: The Legacy of John Percival, Linnean Society, London, pp 37–59

[koac130-B134] Furuta Y, Nishikawa K , Tanino T (1974) Stability in DNA content of AB genome component of common wheat during the past seven thousand years. Jpn J Genet 49: 179–187

[koac130-B1] Ohno S (1970) Evolution by Gene Duplication. Springer, New York, NY

[koac130-B135] Okamoto M (1957) Asynaptic effect of chromosome V. Wheat Inf Serv 5: 6

[koac130-B136] Orth RA , Bushuk W (1973) Studies of glutenin: III. Identification of subunits coded by the D-genome and their relation to breadmaking quality. Cereal Chem 50: 680–687

[koac130-B137] Ozkan H , Brandolini A, Pozzi C, Effgen S, Wunder J, Salamini F (2005) A reconsideration of the domestication geography of tetraploid wheats. Theor Appl Genet 110: 1052–1060 15714326 10.1007/s00122-005-1925-8

[koac130-B138] Ozkan H , Feldman M (2001) Genotypic variation in tetraploid wheat affecting homoeologous pairing in hybrids with *Aegilops peregrina*. Genome 44: 1000–1006 11768203

[koac130-B139] Ozkan H , Levy AA, Feldman M (2001) Allopolyploidy-induced rapid genome evolution in the wheat (Aegilops-Triticum) group. Plant Cell 13: 1735–1747 11487689 10.1105/TPC.010082 PMC139130

[koac130-B140] Peng J , Ronin Y, Fahima T, Röder MS, Nevo E, Korol A (2003) Domestication quantitative trait loci in *Triticum dicoccoides*, the progenitor of wheat. Proc Natl Acad Sci USA 100: 2489–2494 12604784 10.1073/pnas.252763199 PMC151368

[koac130-B141] Percival J (1921) The Wheat Plant: A Monograph. Duckworth, London, pp 1–46

[koac130-B142] Pikaard CS (2000) The epigenetics of nucleolar dominance. Trend Genet 16: 495–500 10.1016/s0168-9525(00)02113-2 11074291

[koac130-B143] Piperno DR , Weiss E, Holst I, Nadel D (2004) Processing of wild cereal grains in the upper Palaeolithic revealed by starch grain analysis. Nature 430: 670–673 15295598 10.1038/nature02734

[koac130-B144] Pont C , Leroy T, Seidel M, Tondelli A, Duchemin W, Armisen D, Lang D, Bustos-Korts D, Goue N, Balfourier F, et al (2019) Tracing the ancestry of modern bread wheats. Nat Genet 51 905–911 31043760 10.1038/s41588-019-0393-z

[koac130-B145] Pourkheirandish M , Hensel G, Kilian B, Senthil N, Chen G, Sameri M, Azhaguvel P, Sakuma S, Dhanagond S, Sharma R, et al (2015) Evolution of the grain dispersal system in barley. Cell 162: 527–539 26232223 10.1016/j.cell.2015.07.002

[koac130-B146] Presting GG , Malysheva L, Fuchs J, Schubert IZ (1998) A *TY3/GYPSY* retrotransposon-like sequence localizes to the centromeric regions of cereal chromosomes. Plant J 16: 721–728 10069078 10.1046/j.1365-313x.1998.00341.x

[koac130-B147] Ramírez-González RH , Borrill P, Lang D, Harrington SA, Brinton J, Venturini L, Davey M, Jacobs J, van Ex F, Pasha A, **et al.** (2018) The transcriptional landscape of polyploid wheat. Science 361: eaar6089 30115782 10.1126/science.aar6089

[koac130-B148] Rey MD , Martín AC, Higgins J, Swarbreck D, Uauy C, Shaw P, Moore G (2017) Exploiting the ZIP4 homologue within the wheat *Ph1* locus has identified two lines exhibiting homoeologous crossover in wheat-wild relative hybrids. Mol Breed 37: 95 28781573 10.1007/s11032-017-0700-2 PMC5515957

[koac130-B149] Rey MD , Martín AC, Smedley M, Hayta S, Harwood W, Shaw P, Moore G (2018) Magnesium increases homoeologous crossover frequency during meiosis in ZIP4 (*Ph1* Gene) mutant wheat-wild relative hybrids. Front Plant Sci 9: 509 29731763 10.3389/fpls.2018.00509 PMC5920029

[koac130-B150] Rice A , Šmarda P, Novosolov M, Drori M, Glick L, Sabath N, Meiri S, Belmaker J, Mayrose I (2019) The global biogeography of polyploid plants. Nat Ecol Evol 3: 265–273 30697006 10.1038/s41559-018-0787-9

[koac130-B151] Riehl S , Zeidi M, Conard NJ (2013) Emergence of agriculture in the foothills of the Zagros Mountains of Iran. Science 341: 65–67 23828939 10.1126/science.1236743

[koac130-B152] Riley R (1960) The diploidization of polyploid wheat. Heredity 15: 407–429

[koac130-B153] Riley R , Chapman V (1958) Genetic control of the cytologically diploid behaviour of hexaploid wheat. Nature 182: 713–715

[koac130-B154] Saintenac C , Falque M, Martin OC, Paux E, Feuillet C, Sourdille P (2009) Detailed recombination studies along chromosome 3B provide new insights on crossover distribution in wheat (*Triticum aestivum* L.). Genetics 181: 393–403 19064706 10.1534/genetics.108.097469 PMC2644935

[koac130-B155] Sakamura T (1918) Kurze Mitteilung uber die chromosomenzahalen und die Verwandtschaftsverhaltnisse der Triticum Arten. Bot Mag Tokyo 32: 151–154

[koac130-B156] Salina EA , Numerova OM, Ozkan H, Feldman M (2004) Alterations in subtelomeric tandem repeats during early stages of allopolyploidy in wheat. Genome 47: 860–867 15499400 10.1139/g04-044

[koac130-B157] Salse J , Bolot S, Throude M, Jouffe V, Piegu B, Quraishi UM, Calcagno T, Cooke R, Delseny M, et al (2008) Identification and characterization of shared duplications between rice and wheat provide new insight into grass genome evolution. Plant Cell 20: 11–24 18178768 10.1105/tpc.107.056309 PMC2254919

[koac130-B158] Sax K (1918) The behavior of chromosomes in fertilization. Genetics 3: 309–327 17245907 10.1093/genetics/3.4.309 PMC1200439

[koac130-B159] Sax K (1921) Chromosome relationships in wheat. Science 54: 413–415 17735074 10.1126/science.54.1400.413

[koac130-B160] Sax K (1922) Sterility in wheat hybrids. II. Chromosome behavior in partially sterile hybrids. Genetics 7: 513–552 17245991 10.1093/genetics/7.6.513 PMC1200543

[koac130-B161] Schultze-Motel J (2019) Triticum parvicoccum Kislev in Transcaucasia. Genet Resour Crop Ev 66: 1363–1366

[koac130-B162] Sears ER (1952) Homoeologous chromosomes in Triticum aestivum. Genetics 37: 624

[koac130-B163] Sears ER (1954) The aneuploids of common wheat. Missouri Agric Exp Stn Res Bull 572: 1–58

[koac130-B164] Sears ER (1959) Wheat cytogenetics. Ann Rev Genet 3: 451–468

[koac130-B165] Sears ER (1976) Genetic control of chromosome pairing in wheat. Ann Rev Genet 10: 31–51 797311 10.1146/annurev.ge.10.120176.000335

[koac130-B166] Sears ER (1977) An induced mutant with homoeologous chromosome pairing. Can J Genet Cytol 19: 585–593

[koac130-B167] Sears ER (1982) A wheat mutation conditioning an intermediate level of homoeologous chromosome pairing. Can J Genet Cytol 24: 715–719

[koac130-B168] Sears ER (1984) Mutations in wheat that raise the level of meiotic chromosome pairing. *In* Gustafson JP (ed) Gene Manipulation in Plant Improvement. Stadler Genetics Symposia Series. Springer, Boston, MA, pp 295–300

[koac130-B169] Sears ER , Okamoto M (1958) Intergenomic chromosome relationships in hexaploid wheat. Proc 10^th^ Intern Congr Genet, Montreal, Quebec 2: 258–259

[koac130-B170] Senerchia N , Felber F, Parisod C (2015) Genome reorganization in F1 hybrids uncovers the role of retrotransposons in reproductive isolation. Proc Biol Sci Royal Soc 282: 20142874 10.1098/rspb.2014.2874 PMC4375867 25716787

[koac130-B171] Serra H , Svacina R, Baumann U, Whitford R, Sutton T, Bartos J, Sourdille P (2021) Ph2 encodes the mismatch repair protein MSH7-3D that inhibits wheat homoeologous recombination. Nat Commun 12: 803 33547285 10.1038/s41467-021-21127-1 PMC7865012

[koac130-B172] Shaked H , Kashkush K, Ozkan H, Feldman M, Levy AA (2001) Sequence elimination and cytosine methylation are rapid and reproducible responses of the genome to wide hybridization and allopolyploidy in wheat. Plant Cell 13: 1749–1759 11487690 10.1105/TPC.010083 PMC139131

[koac130-B173] Shalev G , Levy AA (1997) The maize transposable element Ac induces recombination between the donor site and an homologous ectopic sequence. Genetics 146: 1143–1151 9215915 10.1093/genetics/146.3.1143 PMC1208042

[koac130-B174] Shao Q , Li C (1983) Basang C semi-wild wheat from Xizang (Tibet). *In* S Sakamoto, ed, 6th International Wheat Genetics Symposium, Kyoto, Japan, pp 111–114

[koac130-B175] Sharma JS , Running KLD, Xu SS, Zhang Q, Peters Haugrud AR, Sharma S, McClean PE, Faris JD (2019) Genetic analysis of threshability and other spike traits in the evolution of cultivated emmer to fully domesticated durum wheat. Mol Genet Genomics 294: 757–771 30887143 10.1007/s00438-019-01544-0

[koac130-B176] Sharma S , Schulthess A, Bassi F, Badaeva E, Neumann K, Graner A, Özkan H, Werner P, Knüpffer H, Kilian B (2021) Introducing beneficial alleles from plant genetic resources into the wheat germplasm. Biology 10: 982 34681081 10.3390/biology10100982 PMC8533267

[koac130-B177] Shewry PR (2009) Wheat. J Exp Bot 60: 1537–1553 19386614 10.1093/jxb/erp058

[koac130-B178] Shewry PR , Hey SJ (2015) The contribution of wheat to human diet and health. Food Energy Secur 4: 178–202 27610232 10.1002/fes3.64 PMC4998136

[koac130-B179] Simonetti MC , Bellomo MP, Laghetti G, Perrino P, Simeone R, Blanco A (1999) Quantitative trait loci influencing free-threshing habit in tetraploid wheats. Genet Resource Crop Evol 46: 267–271

[koac130-B180] Simons KJ , Fellers JP, Trick HN, Zhang Z, Tai YS, Gill BS, Faris JD (2006) Molecular characterization of the major wheat domestication gene *Q*. Genetics 172: 547–555 16172507 10.1534/genetics.105.044727 PMC1456182

[koac130-B181] Slotkin RK , Martienssen R (2007) Transposable elements and the epigenetic regulation of the genome. Nat Rev Genet 8: 272–285 17363976 10.1038/nrg2072

[koac130-B182] Smith O , Momber G, Bates R, Garwood P, Fitch S, Pallen M, Gaffney V, Allaby RG (2015) Archaeology. Sedimentary DNA from a submerged site reveals wheat in the British Isles 8000 years ago. Science 347: 998–1001 25722413 10.1126/science.1261278

[koac130-B183] Snir A , Nadel D, Groman-Yaroslavski I, Melamed Y, Sternberg M, Bar-Yosef O, Weiss E (2015) The origin of cultivation and proto-weeds, long before neolithic farming. PLoS One 10: e0131422 26200895 10.1371/journal.pone.0131422 PMC4511808

[koac130-B184] Soltis DE , Soltis PS (1999) Polyploidy: recurrent formation and genome evolution. Trends Ecol Evol 14: 348–352 10441308 10.1016/s0169-5347(99)01638-9

[koac130-B185] Soltis PS , Soltis DE (2009) The role of hybridization in plant speciation. Annu Rev Plant Biol 60: 561–588 19575590 10.1146/annurev.arplant.043008.092039

[koac130-B186] Stebbins GL (1950) Variation and Evolution in Plants. Columbia University Press, New York, NY

[koac130-B187] Stebbins GL (1971) Chromosomal Evolution in Higher Plants. Edward Arnold Ltd, London

[koac130-B188] Su H , Liu Y, Liu C, Shi Q, Huang Y, Han F (2019) Centromere satellite repeats have undergone rapid changes in polyploid wheat subgenomes. Plant Cell 31: 2035–2051 31311836 10.1105/tpc.19.00133 PMC6751130

[koac130-B189] Tanno K , Willcox G (2006) How fast was wild wheat domesticated? Science 311: 1886 16574859 10.1126/science.1124635

[koac130-B190] Tirosh I , Reikhav S, Levy AA, Barkai N (2009) A yeast hybrid provides insight into the evolution of gene expression regulation. Science 324: 659–662 19407207 10.1126/science.1169766

[koac130-B191] Tschermak E (1914) Die Verwertung der Bastardierung für phylogenetische Fragen in der Getreidegruppe. Zeitschr Pflanzenzucht 2: 291–312

[koac130-B192] Tsunewaki K (2009) Plasmon analysis in the *Triticum-Aegilops* complex. Breed Sci 59: 455–470

[koac130-B193] Tzarfati RV. , Barak V, Krugman T, Fahima T, Abbo A, Saranga Y, Korol AB (2014) Novel quantitative trait loci underlying major domestication traits in tetraploid wheat. Mol Breed 34: 1613–1628

[koac130-B194] Valenzuela NT , Perera E, Naranjo T (2013) Identifying crossover-rich regions and their effect on meiotic homologous interactions by partitioning chromosome arms of wheat and rye. Chromosome Res 21: 433–445 23843032 10.1007/s10577-013-9372-x

[koac130-B195] Venske E , Dos Santos RS, Busanello C, Gustafson P, Costa de Oliveira A (2019) Bread wheat: a role model for plant domestication and breeding. Hereditas 156: 16 31160891 10.1186/s41065-019-0093-9 PMC6542105

[koac130-B196] Walkowiak S , Gao L, Monat C, Haberer G, Kassa MT, Brinton J, Ramirez-Gonzalez RH, Kolodziej MC, Delorean E, Thambugala D, et al (2020) Multiple wheat genomes reveal global variation in modern breeding. Nature 588: 277–283 33239791 10.1038/s41586-020-2961-x PMC7759465

[koac130-B197] Wang X , Wang J, Jin D, Guo H, Lee TH, Liu T, Paterson AH (2015) Genome alignment spanning major Poaceae lineages reveals heterogeneous evolutionary rates and alters inferred dates for key evolutionary events. Mol Plant 8: 885–898 25896453 10.1016/j.molp.2015.04.004

[koac130-B198] Watanabe N , Fujii Y, Kato N, Ban T, Martinek P (2006) Microsatellite mapping of the genes for brittle rachis on homoeologous group 3 chromosomes in tetraploid and hexaploid wheats. J Appl Genet 47: 93–98 16682748 10.1007/BF03194606

[koac130-B199] Watson A , Ghosh S, Williams MJ, Cuddy WS, Simmonds J, Rey MD, Asyraf Md Hatta M, Hinchliffe A, Steed A, Reynolds D, et al (2018) Speed breeding is a powerful tool to accelerate crop research and breeding. Nat Plants 4: 23–29 29292376 10.1038/s41477-017-0083-8

[koac130-B200] Wicker T , Gundlach H, Spannagl M, Uauy C, Borrill P, Ramirez-Gonzalez RH, De Oliveira R, Mayer KFX, Paux E, Frederic C (2018) Impact of transposable elements on genome structure and evolution in bread wheat. Genome Biol 19: 103 30115100 10.1186/s13059-018-1479-0 PMC6097303

[koac130-B201] Wicker T , Zimmermann W, Perovic D, Paterson AH, Ganal M, Graner A, Stein N (2005) A detailed look at 7 million years of genome evolution in a 439 kb contiguous sequence at the barley *Hv-eIF4E* locus: recombination, rearrangements and repeats. Plant J 41: 184–194 15634196 10.1111/j.1365-313X.2004.02285.x

[koac130-B202] Willcox G (2012). Pre-domestic cultivation during the late pleistocene and early Holocene in the Northern Levant. *In* Gepts P, Famula T, Bettinger R, Brush S, Damania A, McGuire P, Qualset CO, eds, Biodiversity in Agriculture: Domestication, Evolution, and Sustainability. Cambridge University Press, Cambridge, pp 92–109

[koac130-B203] Wulff BBH , Moscou MJ (2014) Strategies for transferring resistance into wheat: from wide crosses to GM cassettes. Front Plant Sci 5: 692 10.3389/fpls.2014.00692 PMC4255625 25538723

[koac130-B204] Xiong W , Reynolds MP, Crossa J, Schulthess U, Sonder K, Montes C, Addimando N, Singh RP, Ammar K, Gerard B, et al (2021) Increased ranking change in wheat breeding under climate change. Nat Plants 7: 1207–1212 34462575 10.1038/s41477-021-00988-w

[koac130-B205] Yaakov B , Kashkush K (2011) Methylation, transcription, and rearrangements of transposable elements in synthetic allopolyploids. Int J Plant Genomics 2011: 569826 21760771 10.1155/2011/569826 PMC3134107

[koac130-B206] Yaakov B , Kashkush K (2012) Mobilization of Stowaway-like *MITEs* in newly formed allohexaploid wheat species. Plant Mol Biol 80: 419–427 22933118 10.1007/s11103-012-9957-3

[koac130-B207] Yildirim A , Jones SS, Murray TD, Cox TS, Line RP (1995) Resistance to stripe rust and eyespot diseases of wheat in *Triticum tauschii*. Plant Disease 79: 1230–1236

[koac130-B208] Zeder MA (2011) The origins of agriculture in the Near East. Curr Anthropol 52: S221–S235

[koac130-B209] Zeibig F , Kilian B, Frei M (2021) The grain quality of wheat wild relatives in the evolutionary context. Theor Appl Genet 10.1007/s00122-021-04013-8 PMC9729140 34919152

[koac130-B210] Zhang Z , Belcram H, Gornicki P, Charles M, Just J, Huneau C, Magdelenat G, Couloux A, Samain S, Gill BS, et al (2011) Duplication and partitioning in evolution and function of homoeologous *Q* loci governing domestication characters in polyploid wheat. Proc Natl Acad Sci USA 108: 18737–18742 22042872 10.1073/pnas.1110552108 PMC3219148

[koac130-B211] Zhang Z , Gou X, Kun H, Bian Y, Ma XL, Li M, Gong L, Feldman M, Liu B, Levy AA (2020a) Homoeologous exchanges occur through intragenic recombination generating novel transcripts and proteins in wheat and other polyploids. Proc Natl Acad Sci USA 117: 14561–14571 32518116 10.1073/pnas.2003505117 PMC7321986

[koac130-B212] Zhang Z , Li A, Song G, Geng S, Gill BS, Faris JD, Mao L (2020b) Comprehensive analysis of *Q* gene near-isogenic lines reveals key molecular pathways for wheat domestication and improvement. Plant J 102: 299–310 31778224 10.1111/tpj.14624

[koac130-B213] Zhao G , Zou C, Li K, Wang K, Li T, Gao L, Zhang X, Wang H, Yang Z, Liu X, et al (2017) The *Aegilops tauschii* genome reveals multiple impacts of transposons. Nat Plants 3: 946–955. 29158546 10.1038/s41477-017-0067-8

[koac130-B214] Zhou Y , Bai S, Li H, Sun G, Zhang D, Ma F, Zhao X, Nie F, Li J, Chen L, et al (2021) Introgressing the *Aegilops tauschii* genome into wheat as a basis for cereal improvement. Nat Plants 7: 774–786 34045708 10.1038/s41477-021-00934-w

[koac130-B215] Zhou Y , Zhao X, Li Y, Xu J, Bi A, Kang L, Xu D, Chen H, Wang Y, Wang YG, et al (2020) *Triticum* population sequencing provides insights into wheat adaptation. Nat Genet 52: 1412–1422 33106631 10.1038/s41588-020-00722-w

[koac130-B216] Zohary D , Hopf M (2000) Domestication of Plants in the Old World, Ed 3. Oxford Science Publications, Oxford

[koac130-B217] Zohary D , Hopf M, Weiss E (2012) Domestication of Plants in the Old World-The origin and spread of domesticated plants in south-west Asia, Europe and the Mediterranean Basin, Ed 4. Oxford University Press, Oxford, pp 1–243

